# Development of Radiotracers for Imaging of the PD-1/PD-L1 Axis

**DOI:** 10.3390/ph15060747

**Published:** 2022-06-14

**Authors:** Fabian Krutzek, Klaus Kopka, Sven Stadlbauer

**Affiliations:** 1 Department of Translational TME Ligands, Institute of Radiopharmaceutical Cancer Research, Helmholtz Center Dresden-Rossendorf, 01328 Dresden, Germany; f.krutzek@hzdr.de (F.K.); k.kopka@hzdr.de (K.K.); 2 School of Science, Faculty of Chemistry and Food Chemistry, Technical University Dresden, 01069 Dresden, Germany; 3 German Cancer Consortium (DKTK), Partner Site Dresden, 01307 Dresden, Germany; 4 National Center for Tumor Diseases (NCT), Partner Site Dresden, University Cancer Cancer (UCC), 01307 Dresden, Germany

**Keywords:** tumor microenvironment, PD-1/PD-L1 targeting radiotracer, immune checkpoint, molecular imaging

## Abstract

Immune checkpoint inhibitor (ICI) therapy has emerged as a major treatment option for a variety of cancers. Among the immune checkpoints addressed, the programmed death receptor 1 (PD-1) and its ligand PD-L1 are the key targets for an ICI. PD-L1 has especially been proven to be a reproducible biomarker allowing for therapy decisions and monitoring therapy success. However, the expression of PD-L1 is not only heterogeneous among and within tumor lesions, but the expression is very dynamic and changes over time. Immunohistochemistry, which is the standard diagnostic tool, can only inadequately address these challenges. On the other hand, molecular imaging techniques such as positron emission tomography (PET) and single-photon emission computed tomography (SPECT) provide the advantage of a whole-body scan and therefore fully address the issue of the heterogeneous expression of checkpoints over time. Here, we provide an overview of existing PET, SPECT, and optical imaging (OI) (radio)tracers for the imaging of the upregulation levels of PD-1 and PD-L1. We summarize the preclinical and clinical data of the different molecule classes of radiotracers and discuss their respective advantages and disadvantages. At the end, we show possible future directions for developing new radiotracers for the imaging of PD-1/PD-L1 status in cancer patients.

## 1. Introduction

**Figure 1:**
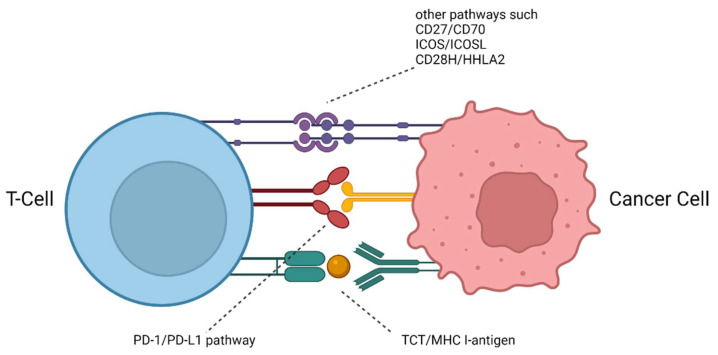
The PD-1/PD-L1 axis within the tumor microenvironment.

The tumor microenvironment (TME) surrounding and supporting cancer cells is a very dynamic immunosuppressive network consisting of different immune cells, such as B cells, T cells, and natural killer (NK) cells, vascular endothelial cells, an extracellular matrix, and cancer-associated fibroblasts (CAFs). Within that network, adipocytes, regulatory T (Treg) cells, fibroblasts, macrophages, and secreted cytokines promote cellular proliferation in all stages of cancer [1]. Therefore, these cellular components represent interesting targets for assessing the status and the therapy of cancers. Additionally, they can serve as imaging biomarkers for the early detection of tumor disease and the monitoring of treatment response. Within the TME, the immune checkpoints play an important role in the evasion of tumor cells from an immune response, which would lead to the recognition and, ultimately, to the elimination of malignant cells. In order to suppress an immune response, tumor cells overexpress certain immune checkpoint proteins to provide a stop signal to the immune system. Among the most important checkpoint proteins are the cytotoxic T-lymphocyte associated protein 4 (CTLA-4, CD152) and the programmed cell death protein 1 (PD-1, CD279), with its corresponding ligand programmed death ligand 1 (PD-L1, CD274) (Figure 1). PD-1 is expressed by CD4+ and CD8+ T cells, regulatory T cells, B cells, and NK cells. Through the interaction with its two ligands, PD-L1 and PD-L2, PD-1 is inactivated. This allows healthy cells, but also tumor cells, to evade an immune attack. While PD-L2 is expressed mainly by dendritic cells, macrophages, and B and Th2 cells, PD-L1 is expressed by T and B cells, macrophages, dendritic cells, epithelial cells, stromal cells, endothelial cells, and tumor cells. Therefore, PD-L1 has become a key target for the development of so-called immune checkpoint inhibitor (ICI) therapy, a promising cancer treatment option [2]. Monoclonal antibodies (mAb) directed either towards PD-1 or PD-L1 are used as therapeutics, which, upon binding to the immune checkpoints, prevent their blockade and thus lead to the reactivation of the innate immune response. The use of such immune checkpoint inhibitors has resulted in the successful treatment of a variety of cancers, leading to the improved survival rates of patients [3,4,5,6,7]. However, on average, only one out of three patients respond to an immune checkpoint inhibitor monotherapy [8,9,10]. In order to identify those patients most likely to respond prior to therapy, molecular imaging techniques such as PET and SPECT are advantageous and can complement classical immunohistochemistry methods because they are able to visualize the whole body [11,12]. Thus, the issue of the heterogeneous expression of these immune checkpoints within and across tumor lesions, as well as their dynamic changes in upregulation over time, could be addressed. In addition, molecular imaging is non-invasive and would greatly reduce the burden for patients, who otherwise have to undergo repeated biopsies. Radiolabeled large (e.g., antibodies, antibody-fragments, minibodies, and affibodies) and small (e.g., peptides, peptidomimetics, and non-peptides) molecules are therefore very attractive as radiotracers for the imaging of PD-1 or PD-L1 up- and dysregulation in cancer patients, because they could support therapy decisions and help to monitor the success of an ongoing therapy. Here, we provide an overview of the different molecule classes of radiotracers for PD-1 and PD-L1 imaging. At the end, we will discuss the advantages and disadvantages of each class with respect to their clinical application and provide future directions for the development of improved radiotracers for immune checkpoint imaging.

## 2. Results

In the past 10 years, a large number of radioligands based on antibodies, mono- and minibodies, and affibodies and nanobodies have been developed and investigated both preclinically and clinically for the imaging of either PD-1 or PD-L1 up- and dysregulation—or both simultaneously—in cancer patients. More recently, peptides derived from the inhibitor screening of pharmaceutical companies have been transformed into radioligands for the imaging of PD-L1 but not PD-1 and have been studied extensively in preclinical settings. Additionally, within the past two years, the first small molecule-based radiotracers for the imaging of PD-L1 expression have been reported. However, thus far, in vivo studies have been limited. The following Table 1 provides an overview of the reported imaging agents for PD-1 and PD-L1 sorted by decreasing molecular weights. We applied the official nomenclature rules for radioactive compounds published in 2017 by Coenen et al. and, therefore, standardized the names of the radiotracers mentioned in this article [13]. 

**Table 1:** Overview imaging agents addressing PD-1 and PD-L1 classified by substance name, imaging agent, intended application, and binding affinity (K_D_/EC_50_).

Target	Class	Substance Name	Imaging Agent	Intended Application	K_D_/EC_50_ [nM]	Ref.
PD-1	mAb	DOTA-anti-mouse-PD-L1	^64^Cu	PET	n.a.	[14]
	mAb	JS001	^99m^Tc	SPECT	n.a.	[15]
	mAb	Df-Nivolumab	^89^Zr	PET	3.75 nM	[16,17,18]
	mAb	Df-Pembrolizumab	^89^Zr	PET	n.a.	[19,20]
		Keytruda	^64^Cu ^89^Zr	PET	n.a.	[21,22]
		N-sucDf-Pembrolizumab	^89^Zr	PET	n.a.	[23]
	mAb	PD-1-Liposome-DOX	^64^Cu IRDye800CW	PET NIRF	n.a.	[24]
	mAb	PD-1-IRDye800CW	IRDye800CW	NIRF	n.a.	[25]
PD-1/PD-L1	mAb	NOTA-α-PD-1 (RMP1-14) NOTA-α-PD-L1 (10F.9G2)	^64^Cu	PET	n.a.	[26]
	mAb Adnectin	Df-Nivolumab BMS-986192	^89^Zr [^18^F]AlF	PET	n.a.	[18]
PD-L1	mAb	PD-L1.3.1	^111^In	SPECT	0.97 nM	[27]
	mAb	DTPA-anti-PDL1	^111^In	SPECT	0.6 ± 0.1 nM	[28,29]
	mAb	anti-mPD-L1	^111^In	SPECT	1.1 ± 0.1 nM	[30]
	mAb	C4	^89^Zr	PET	4.2 ± 0.7 nM 1.5 ± 0.34 nM	[31]
	mAb	Avelumab	^89^Zr	PET	0.3 nM	[32,33]
	mAb	Atezolizumab	Licor 800	NIRF	0.43 nM ^2^ (human) 0.13 nM ^1^ (mouse)	[34,35]
			^111^In	SPECT		
			^64^Cu	PET		[36]
			^89^Zr			[37,38,39]
			^99m^Tc	SPECT	111.8 ± 17.85 nM	[40]
	mAb	NOTA-MX001	^64^Cu	PET	5.40 ± 2.30 nM	[41]
	mAb	[^89^Zr]Zr-DFO-anti-PD-L1 mAb	^89^Zr	PET	n.a.	[42]
	mAb	Df-KN035	^89^Zr	PET	2.86 ± 0.23 nM	[43,44]
	mAb	PD-L1-Mab	^131^I	Cherenkov Luminescence	1.069 nM	[45]
	mAb	PD-L1 mAb	^131^I	Optical	n.a.	[46]
	mAb	NIR-PD-L1-mAb	Licor 800	NIRF	n.a.	[47]
	HCAb	anti-hPD-L1 Nb6	^124^I ^125^I	PET	2.19 nM	[48]
		NOTA-Nb6	^64^Cu	PET	3.60 nM	[49]
	Fab Fragment	NOTA-αPD-L1	^64^Cu	PET	0.72 nM (EC_50_)	[50]
	Nanobody	NOTA-Nb109	^68^Ga	PET	2.9 nM	[51]
	Nanobody	C3 C7 E2 E4	^99m^Tc	SPECT	0.5 nM 17.0 nM 2.1 nM 4.0 nm	[52]
	Nanobody	NOTA-(hPD-L1)	^68^Ga	PET	0.8 nM 1.2 nM	[53]
	Nanobody	NM-01	^99m^Tc	SPECT	n.a.	[54]
	Affibody	NOTA-Z_PD-L1_1_	^18^FAl	PET	1 nM	[55]
	Affibody	NOTA-Z_PD-L1_4_	^18^FAl ^68^Ga	PET	0.07 nM	[56]
PD-L1	Peptide	[^64^Cu]Cu-WL12	^64^Cu	PET	2.9 nM	[57]
		[^68^Ga]Ga-WL12	^68^Ga	PET	n.a.	[58]
		[^18^F]FPy-WL12	^18^F	PET	37.1 nM	[59]
	Peptide	AlF-TPP-1 AlF-PEG-TPP-1	^18^F	PET	95 nM ^2^ [60]	[61]
		TPP-1 PEG-TPP-1	^64^Cu			
		[^18^F]AlF-NOTA-IPB-PDL1P	^18^F	PET	n.a.	[62]
		[^68^Ga]Ga-NJMP1	^68^Ga	PET	25.9 µM	[63]
	Small Molecule	[^18^F]FLN	^18^F	PET	65.3 nM	[64]
		[^18^F]FLG-1	^18^F		63.1 nM	[65]
	HAC-PD1	DOTA-HAC	^64^Cu	PET	~110 pM	[66]
		NOTA-HAC NOTA-HACA DOTA-HACA	^64^Cu ^68^Ga	PET	~110 pM	[67]
	Engineered Protein	FN3_hPD-L1_	^64^Cu	PET	1.4 ± 0.3 nM	[68]
	Adnectin	BMS-986192	[^18^F]AlF	PET	<35 pM	[18,69,70,71]
	Nanoparticle	αPDL1-GNP	GNP	CT	n.a.	[72]

^1^ Values for non-conjugated Atezolizumab. ^2^ Value for unsubstituted peptide; K_D_ for substituted peptide was found to be in the same range.

In the following chapters, the different radiotracers of each molecular class will be discussed and compared with regard to their in vitro and in vivo and preclinical and clinical results. At the end, the different classes will be compared, and their advantages and disadvantages will be discussed. 

### 2.1. PD-1-Targeting Radiotracers

#### Antibodies

Full monoclonal antibodies (mAbs)–proteins with molecular weights of about 150 kDa–consist of two heavy chains (50 kDa) and two light chains (25 kDa). The antigen binding fragment (Fab) contains the complementarity-determining region, which leads to highly specific antigen binding with possible binding constants in the femtomolar range. However, their high molecular weight results in slow biodistribution and low tissue penetration. Therefore, PET or SPPECT imaging is usually possible four to seven days after tracer administration to obtain suitable images [73,74]. 

Natarajan et al., in 2015, were the first to report on an antibody-based tracer for imaging PD-1 by PET/CT [14]. An anti-mouse-PD-1 monoclonal antibody was coupled with DOTA-NCS at its lysine sites for radiolabeling with copper-64, and the binding to PD-1 was confirmed by FACS experiments. Treg cells, known to express PD-1, were inoculated in melanoma tumor-bearing mice, and bioluminescence images (BLI) were acquired 5 min after the injection of d-luciferin. The tumor and spleen both exhibited high luminescence counts in comparison to other tissues. PET/CT scans were acquired, and ex vivo biodistribution revealed the highest tumor uptake after 24 h p.i. (9.37 ± 0.09 %ID/g, tumor-to-muscle ratio of 11.0, tumor-to-blood ratio of 0.8). However, high activity in the liver and spleen was detected, which decreased slightly after 48 h p.i.. Blocking studies proved the specificity of [^64^Cu]Cu-DOTA-anti-mouse-PD-1, and ex vivo analysis revealed a reduced uptake in the tumor and spleen, accordingly. 

Guo et al. labeled the recombinant humanized monoclonal antibody JS001 by its reaction with Na[[^99m^Tc]TcO_4_] [15]. The micro-SPECT images acquired in tumor-bearing mice at several time points (3, 8 and 28 h) showed the delineation of the tumor, with the highest contrast at 28 h p.i.. The biodistribution at this time point showed that [^99m^Tc]Tc-JS001 accumulated in both the kidneys and liver, while it did so only moderately in the tumor. High blood pool activity at 28 h p.i., however, indicated the incomplete enrichment of the radiotracer.

Nivolumab—a full human IgG4 anti-PD-1 monoclonal antibody—is currently undergoing clinical trials for immune checkpoint inhibitor therapy. Hence, Cole et al. modified nivolumab with desferrioxamine (DFO) and tested the affinity of nivolumab and DFO-nivolumab in vitro (K_D_ = 3.10 and 3.75 nM, respectively) [16]. [^89^Zr]Zr-DFO-nivolumab and carrier-added [^89^Zr]Zr-DFO-nivolumab (1 and 3 mg/kg of nivolumab) were injected in normal healthy cynomolgus monkeys to study the biodistribution by PET imaging. The splenic uptake of the tracer was high only at days 4, 6, and 8 (SUVs of approx. 16.7–17.7), but it was reduced by 83% to 87% for carrier-added tracer applications (for both 1 and 3 mg/kg administrations) at each time point, indicating the specificity of [^89^Zr]Zr-nivolumab due to natively expressed dendritic cells in the spleen. However, no uptake was observed for the no-carrier-added tracer (SUV of approx. 4–6 for all time points) due to the liberation of ^89^Zr from the chelator. Since antibodies are mainly excreted by the liver, a certain hepatobiliary uptake was detected. The uptake in the other non-target tissues was low regardless of the co-administered amounts of unlabeled nivolumab. In a separate study, England et al. conducted in vivo studies using ^89^Zr-labelled DFO-nivolumab in two different mouse models: immunocompetent and immunodeficient mice both bearing A549 tumors in their lower flank [17]. By the ROI analysis of the acquired PET images, the tumors were already delineated at 6 h p.i. in both models but showed differences in terms of visualization at later time points: The tumor uptake for NSG mice peaked at 24 h p.i., whereas for PBL mice, the accumulation in the tumor gradually increased up to 168 h p.i. The tracer was similarly distributed in the blood pool and in non-target tissues over all time points for both mouse models (range of approx. 6–8 %ID/g in liver, kidney, spleen at 48 h p.i. for both models). The specificity of the [^89^Zr]Zr-nivolumab uptake was verified in comparison with a non-specific [^89^Zr]Zr-DFO-IgG control antibody.

PD-1-targeting Pembrolizumab (commercial name Keytruda)—a humanized IgG4 monoclonal antibody—showed success in the treatment of advanced melanoma, non-small cell lung cancer, and other malignancies [75]. This inspired the same group to utilize ^89^Zr-labelled DFO-pembrolizumab for PD-1 imaging [19]. Over the whole-time span, the liver uptake decreased by 50%, whereas the spleen uptake was only reduced by 20%. Ex vivo biodistribution (7 d p.i.) confirmed these results. Similar experiments were conducted with rats, showing a fourfold decrease in the activity of organs between 0.5 h and 7 d p.i. For both mouse and rat experiments, moderate bone uptakes were observed, which contributed to the liberation of ^89^Zr from the chelator. A humanized mouse model of human peripheral blood lymphocytes (PBLs) in severe combined immunodeficient NSG mice showed a reduced blood circulation time and a significantly higher uptake in the salivary glands compared to those for immunodeficient NSG mice.

Van der Veen et al. optimized the conjugation of pembrolizumab with the TFP-N-sucDf chelator and its radiolabeling with ^89^Zr [20]. Compared to the study by England et al., the mice were additionally administered a non-specific [^89^Zr]Zr-IgG_4_ antibody, revealing non-significantly lower uptakes in the tumors compared to [^89^Zr]Zr-sucDf-Pembrolizumab, which consequently led to similar tumor-to-blood ratios. The higher uptake in lymphoid tissues partly indicated at least the PD-1 specificity binding of [^89^Zr]Zr-sucDf-Pembrolizumab. A blocking experiment by co-administering a 10-fold excess of unlabeled pembrolizumab led to reduced uptake in the spleen, other lymphoid organs, and the liver, while the activity in the blood pool increased, but the tumor uptake remained unaffected.

**Figure 2:**
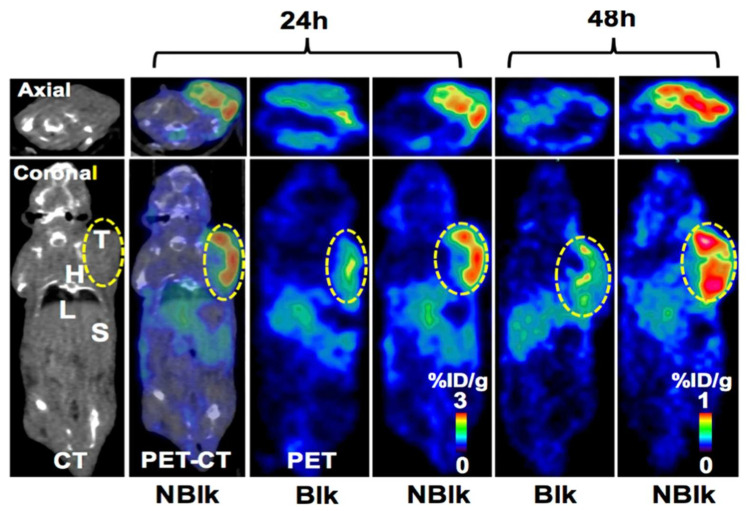
PET/CT image showing [^64^Cu]Cu-DOTA-Pembrolizumab immunoPET in the hNSG/A375 mouse model. Representative PET/CT axial and coronal images displayed at 24 and 48 h post-injection of tracer (7.4 MBq/200 μL) in hNSG/A375-blk and hNSG/A375-nblk mice. T, tumor; H, heart; L, liver; S, spleen. Adapted with permission from [22].

Natarajan et al. modified Pembrolizumab with a DFO- and DOTA-chelator for ^89^Zr and ^64^Cu labeling, respectively [21]. The resulting [^89^Zr]Zr-DFO-Keytruda was studied in NSG mice bearing a human peripheral blood mononuclear cell (hNSG-nblk mice) tumor (Figure 2). The splenic uptake was similar in comparison to the mouse control group (NSG-ctl, mice with no engrafted PBLs) at 24 h, but the tumor-to-muscle ratios were significantly higher for the hNSG-nblk mice. The liver uptake was already low at 24 h and dropped further at 144 h for the hNSG-nblk mice. PET/CT images of [^64^Cu]Cu-DOTA-Keytruda acquired at 4 and 24 h p.i. showed increasing tumor uptakes in the NSG-ctl, hNSG-blk (blocking group) and hNSG-nblk groups at 24 h. A similar trend was observed for the spleen uptake and the tumor-to-muscle ratios. Overall, [^64^Cu]Cu-DOTA-Keytruda showed better in vivo results compared to [^89^Zr]Zr-DFO-Keytruda and thus may be the better radiotracer for PET imaging at early time points. Additionally, the lower manufacturing costs of ^64^Cu are an advantage over ^89^Zr, and DOTA is well profiled for its toxicity in clinical settings. However, copper is known to be unstable in DOTA, leading to transchelation in the liver [76]. In a separate study, Natarajan et al. published a dosimetry study for [^64^Cu]Cu-DOTA-Pembrolizumab [22]. By ex vivo immunoPET biodistribution, the tracer uptakes for each tissue were calculated and revealed the highest radiation burdens for non-blocked mice in the liver (34.2 ± 3.4 µSv/MBq) and red marrow (24.1 ± 0.3 µSv/MBq). Based on these results, human equivalent doses were estimated, predicting that the liver will be the dose-limiting organ (33–38 µSv/MBq).

Li et al. evaluated the biodistribution of ^89^Zr-labelled pembrolizumab in healthy cynomolgus monkeys [23]. At days 0 and 2, uptake was predominantly observed in primary and secondary lymphoid organs—to some extent, in the heart, liver, and kidney—whereas accumulation in the muscle and brain was low and decreased further during the seven-day course. At the latest time point, the highest tissue-to-blood pool ratios were observed in the spleen, in the mesenteric lymph nodes (5.9), and in the tonsils (2.6) for the first animal. For the monkey, which received unlabeled pembrolizumab prior to the tracer injection, an increased activity in the blood pool, lower lymphoid tissue-to-blood ratios, and similar non-lymphoid tissue-to-blood ratios were noted in comparison to the other subjects. These results indicate the PD-1 mediated uptake of [^89^Zr]Zr-N-sucDf-pembrolizumab in lymphoid tissues. Based on these animal experiments, the human organ-absorbed radiation was estimated. The highest doses were found in the testes and in the spleen for the male and female monkeys, respectively. Quite high absorbed doses were also calculated for the liver and kidneys. The whole-body effective dose was estimated for an adult human (0.88 ± 0.15 mSv/MBq) and was therefore in a similar range compared to other ^89^Zr-labeled antibodies [23,77].

Du et al. pursued a dual-labeled theranostic approach for the imaging of PD-1 with ^64^Cu for PET and with IRDye800CW for the optical imaging and therapy of tumors (using cytostaticum doxorubicin, DOX) by the incorporation of these regimens in liposome nanoparticles [24]. Therefore, the DOTA chelator IRDye800CW and the anti-mouse PD-1 monoclonal antibody RMP1-14 (rat IgG2a antibody) were each conjugated to the fatty acid 1,2-distearoyl-*sn*-glycero-3-phosphorylethanolamine (DSPE), and a liposome suspension was generated, which was subsequently incubated with DOX. The imaging of the liposomes via TEM delineated spherical and homogenous liposomes. In vitro experiments on the breast cancer cell line 4T1-fLuc revealed that, compared to free DOX, both liposomes decreased the survival rate and increased the inhibition rate at 1 and 10 µg/mL. Ex vivo bioluminescence imaging (BLI) proved that liposome-encapsulated DOX retained its cytotoxicity. Fluorescence imaging (FMI) revealed the most significant difference (2.4-fold) in fluorescence at 24 h p.i. for the PD-1 specific liposome, which was further confirmed by ex vivo analysis. PET images 12 and 24 h after injection showed 1.4 and 1.8 higher tumor-to-muscle ratios for [^64^Cu]Cu-DOTA-PD-1-Liposome-DOX compared to its non-specific analogue, respectively. The tumor growth was monitored for 15 days and revealed tumor size decline and improved survival, especially when using the specific PD-1 liposome.

Additionally, Du et al. reported that IRDye800CW conjugated to the lysine residues of a PD-1 monoclonal antibody for fluorescence image-guided surgery [25]. Bioluminescence and fluorescence imaging in tumor-bearing mice showed the delineation of the tumor within 30 min p.i. The highest tumor-to-muscle ratio of 8:1 was observed at 8 h p.i., which was a 1.7-fold increase compared to a non-specific conjugated IgG antibody labeled with IRDye800CW. Additionally, the potential of PD-1-IRDye800CW for real-time intraoperative guidance was studied. A total of 24 h after the tracer injection, the tumors were dissected using a stereo fluorescence microscope. In contrast to traditionally performed surgery, the tumor was completely removed, demonstrating that their conjugate delineates the tumor efficiently and improves the precision of tumor resection. The overall survival of the mice operated under fluorescence image guidance was significantly higher compared to that of the mice receiving normal surgery. 

### 2.2. Combined Studies of PD-1 and PD-L1-Targeting Radiotracers

Hettich et al. combined immunoPET/CT imaging studies of PD-1 and PD-L1 with therapeutical experiments [26]. The αPD-1 (RMP1-14) and PD-L1 (10F.9G2)-targeting monoclonal antibodies were both modified with NOTA to enable ^64^Cu labeling. PET/CT imaging with [^64^Cu]Cu-NOTA-PD-1 in tumor bearing mice after 24 h p.i. delineated the tumor, the B and myeloid cells, and the spleen and individual lymph nodes despite the low expression of PD-1. Besides accumulation in the liver, activity was mainly observed in the blood pool. In contrast to PD-1, PD-L1 is more broadly expressed under physiological conditions on both the hematopoietic and non-hematopoietic cells. PET/CT images acquired 24 h after the injection of [^64^Cu]Cu-NOTA-PD-L1 in mice revealed uptake in the spleen, lymph nodes, and brown adipose fat tissue. In another experiment, tumor-bearing mice were treated with a combined immunoradiotherapy to study the possible induction of tumor-infiltrating lymphocytes (TILs). PET/CT images of immunoradiotherapy-treated mice acquired 24 h after the injection of [^64^Cu]Cu-NOTA-PD-1 clearly delineated the tumor. Mice with wild type and PD-L1-deficient melanomas were administered [^64^Cu]Cu-NOTA-PD-L1 to investigate the differences in PD-L1 uptake. Upon INF-γ treatment, the wildtype tumors exhibited a higher tracer uptake in comparison to the knockout tumors. 

### 2.3. PD-L1-Targeting Radiotracers

#### 2.3.1. Antibodies

Heskamp et al. selected the murine monoclonal IgG1 antibody PD-L1.3.1, which specifically addresses human but not murine PD-L1, for conjugation with DTPA [27]. SPECT/CT imaging of tumor-bearing mice showed that the maximum tumor uptake was achieved after 3 p.i., while the tumor-to-blood ratio peaked at day 7 and remained at that level compared with the mice receiving an excess of a co-injected unlabeled antibody. The uptake in non-target tissues, such as the liver and lungs, remained moderate over the seven-day time period. [^111^In]In-PD-L1.3.1 was also able to discriminate between high and low levels of PD-L1 expression in the tumors. 

One of the first SPECT-based imaging tracers for PD-L1 was developed by Josefsson et al. by the conjugation of DTPA with a murine anti-PD-L1 antibody [28]. In vitro evaluation of [^111^In]In-DTPA-anti-PD-L1 showed a high affinity to PD-L1 (K_D_ = 8.3 ± 3.2 nM). SPECT imaging of tumor-bearing mice showed accumulation in PD-L1-rich sites at 24 and 72 h p.i.. Ex vivo biodistribution showed high activity in the blood pool and a decent uptake in the tumor at 24 h p.i.. The radiolabeled antibody was enriched in both the spleen and the liver, while at 72 h p.i., the accumulation in the tumor peaked, and moderate tumor-to-muscle/blood ratios were noted. Accumulation in the thymus was observed at all of the time points, too. Based on these results, a similar anti-PD-L1 antibody was conjugated with DTPA, labeled with ^111^In, evaluated in vitro, and assessed in vivo in tumor-bearing mice [29]. Interestingly, in blocking experiments, they observed a maximum tumor uptake at a dose of 3 mg/kg, because the spleen served as a sink for the cold antibody occupying the PD-L1-positive sites, thus allowing the radiotracer to pass through the spleen, leading to a higher tumor uptake.

For the PET imaging of PD-L1, Heskamp et al. conjugated a rat IgG2b anti-murine PD-L1 (anti-mPD-L1) antibody with DTPA for labeling with ^111^In [30]. The highest tumor uptake was noted at 3 days p.i. with 30 µg of antibody. Clearance from the tumor and blood was observed at day 7 post-injection. The determination of the imaging sensitivity of ^111^In-anti-mPD-L1 showed a positive correlation between the tumor uptakes and PD-L1 expression levels in tumors, with varying PD-L1 expressions. PD-L1-positive tumors were irradiated, and one day later, [^111^In]In-anti-mPD-L1 was injected to monitor possible PD-L1 upregulation resulting from the radiation. Indeed, a significant increase in PD-L1 expression was noted for the irradiated tumors in comparison to that of the non-irradiated tumors, whereas such an upregulation was not observed for lower expressing PD-L1 xenografts.

To fill the gap between the preclinical studies and the clinical translation, Truillet et al. created a patient-derived xenograft (PDX) [31]. Therefore, they designed an anti-PD-L1 antibody (named C4) by Fab phage display which exhibited high binding affinities to human and murine PD-L1 (EC_50_ values of 9.9 and 5.2 nM, respectively). ^89^Zr-labeled C4 showed accumulation in the tumor, peaking at 48 h p.i., but also showed substantial accumulation in the liver, kidneys, and spleen. PET/CT imaging of mice inoculated with the cells of a PD-L1-positive NSCLC lesion from a patient who was treated with pembrolizumab and ipilimumab 7 months prior showed a low tumor-to-blood ratio (approx. 1.6) in the tumor. To meet the requirements for clinical applications, a tracer has to be capable of competing against the PD-1 antigen in an immunocompetent tumor microenvironment. Therefore, the authors performed biodistribution studies in immunocompetent mice bearing PD-L1-positive tumors. As a result, the tumor uptake peaked at 48 h p.i. (approx. 14 %ID/g), with significant uptakes in the liver and spleen (approx. 7 and 6 %ID/g, respectively). Since chemotherapies/radiation therapies can influence the PD-L1 expression [78,79], a cohort of tumor-bearing mice (nu/nu mice) was treated with paclitaxel and doxorubicin before [^89^Zr]Zr-C4 was administered. PET/CT imaging showed a higher and lower tumor uptake for the mice treated with paclitaxel and doxorubicin, respectively, compared to the vehicle.

These results inspired Li et al. to continue the investigation of the possible clinical translation of [^89^Zr]Zr-Df-Avelumab [32]. ROI analysis of acquired PET/CT images (1–5 days) showed that the tumor uptake peaked in the non-block study at day 2 and declined constantly until day 5. The uptakes in the lymph nodes and spleen were considerably lower compared to those found in the work of Truillet (approx. 7 and 10 %ID/g for all time points, respectively). The blocking studies showed that the uptakes were translocated from the lymph nodes and spleen (approx. 6 and 4 %ID/g for all time points, respectively) to the liver (ranging from approx. 7 (targeting) to 12 (blocking) %ID/g for all time points). These findings indicate a saturation of PD-L1 receptors in the lymph nodes and spleen and an enhanced clearance by the liver. In summary, [^89^Zr]Zr-Df-Avelumab is a promising candidate for clinical translation, and this is supported by the fact that a clinical study of avelumab in immune checkpoint therapy for NSCLC patients is in preparation [80].

Jagoda et al. conjugated avelumab with DFO for labeling with ^89^Zr [33]. A cell saturation assay proved the high binding affinity of PD-L1 with a K_D_ of 0.392 ± 0.0481 nM. PET/CT imaging showed a clearance (except for the femurs and lymph nodes) from non-target tissues. The accumulation in the femurs was considered as a consequence of the liberation of ^89^Zr from the chelator. The spleen and lymph nodes—known as PD-L1 expressing organs—showed the highest tissue uptakes from day 1 to day 7. The tumor uptake remained constant from day 1 to day 7, with a peak at day 5. The biodistribution studies showed a dose-dependent displacement of the tracer by unlabeled avelumab: at the highest co-injected amount, a 7.1-fold decrease in the splenic uptake was achieved. In contrast, the tissue uptakes significantly increased for the tumor and lymph nodes (2- to 5-fold for injected doses of 20, 40, and 400 µg). These increased uptakes are most likely a result of the saturation of natively expressed PD-L1 receptors in the spleen, leading to a higher availability in the blood, which became evident by the 12.7-fold higher uptake in the blood pool. 

Atezolizumab (MPDL3280)—a humanized monoclonal IgG1k antibody—exhibits high affinities to human and murine PD-L1 (K_D_ = 0.43 and 0.13 nM, respectively) and succeeded in clinical trials treating several kinds of cancers including non-small cell lung carcinoma, renal cell carcinoma, and triple negative breast cancer [81,82]. 

**Figure 3:**
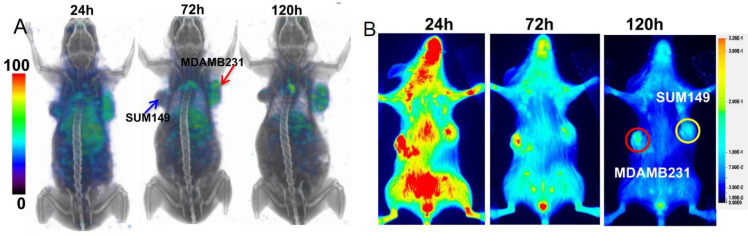
Imaging PD-L1 expression in orthotopic breast cancer xenografts with [^111^In]In-atezolizumab and NIR-atezolizumab. (**A**) NSG mice with orthotopic MDAMB231 and SUM149 xenografts were administered intravenously with 14.8 MBq (400 μCi) of [^111^In]In-atezolizumab or 22 µg of NIR-atez, and images were acquired at 24, 72, and 120 h after the injection of the mAbs. 3D volume-rendered whole-body SPECT/CT images demonstrate the specific accumulation of activity in the MDAMB231 tumors. (**B**) Optical images acquired in the 800 nm NIR channel show the specific accumulation of the fluorescence signal in the MDAMB231 tumors. Adapted with permission from [34].

These promising clinical results inspired Chatterjee et al. to design an atezolizumab-based radiotracer [21]. After conjugation with diethylenetriaminepentaacetic acid (DTPA), this precursor was successfully labeled with ^111^In. In mice bearing a PD-L1-positive and a PD-L1-negative tumor, SPECT/CT imaging showed tracer uptake in the lungs, liver, and spleen (Figure 3). A protein-dose escalation biodistribution experiment showed that the co-injection of 30 µg of an unlabeled antibody provided—via the saturation of PD-L1-positive receptors occurring in the spleen—the best results, with the highest tumor uptake at 48 h p.i., a tumor-to-muscle ratio of 21.71 ± 1.28, and a tumor-to-blood ratio of 2.51 ± 0.11. A moderate uptake in the liver and spleen was observed. Besides radiolabeling, atezolizumab was also conjugated with the near-infrared dye Licor800, and fluorescence imaging and ex vivo biodistribution were carried out. These experiments corroborated the results obtained by the SPECT/CT imaging and ex vivo analysis. Taken together, these findings validated the previous results and underline the specificity of [^111^In]In-PD-L1-atezolizumab for the non-invasive imaging of PD-L1 [34,35]. 

With regard to clinical relevance, Lesniak et al. substituted the radionuclide ^111^In for the more clinically used ^64^Cu to allow for PET imaging. Therefore, they conjugated atezolizumab with DOTAGA [36]. [^64^Cu]Cu-DOTAGA-atezolizumab showed specific in vitro binding on cell lines with varying PD-L1 expression levels. In a mouse xenograft model bearing a PD-L1-positive and PD-L1-negative tumor, PET/CT imaging revealed higher accumulation in the PD-L1-positive tumor compared to that in the PD-L1-negative tumor and the other non-target tissues at 24 and 48 h after injection. Accumulation in the peripheral organs such as the liver, spleen, heart, lungs, kidneys, and thymus were in the same range as that for the PD-L1-negative tumor. Similar experiments in a tumor model with a lower PD-L1 expression and a mammary tumor model of immunocompetent mice corroborated these results.

^89^Zr-labeled atezolizumab was also investigated by Ehlerding et al. for the PET imaging of tumor bearing mice with regard to the influence of radiotherapy upon PD-L1 upregulation [39]. [^89^Zr]Zr-Df-atezolizumab was then injected in mice bearing PD-L1-positive tumors, and PET images were acquired, which showed high accumulation in the spleen and lymph nodes. The tumor uptake peaked at 24 h p.i. for the non-irradiated mice, which was similar for the group receiving a single dose of 5 Gy. For the group receiving five times 2 Gy, the tumor uptake was higher. The tumor-to-muscle ratios peaked at 96 h p.i. for the non-irradiated, the 5 Gy × 1 Fx, and the 2 Gy × 5 Fx groups, respectively, proving a high circulation time of [^89^Zr]Zr-Df-atezolizumab in the blood pool. The irradiated PD-L1-negative xenografts did not exhibit any increase in tracer uptake upon radiation but showed nearly identical uptakes compared to the PD-L1-positive tumors, which could be attributed to enhanced permeability retention effects and natively expressed levels of PD-L1 on this cell line. 

Atezolizumab was also used by Qui et al., who designed a SPECT tracer with the radionuclide ^99m^Tc, using a pre-targeting approach [40]. They attached a *trans*-cyclooctyne (TCO) moiety to atezolizumab via NHS-conjugation and synthesized a tetrazine-based precursor ([^99m^Tc]TcHYNIC-PEG11-Tz) which underwent—after labeling with ^99m^Tc–a copper—a free click reaction with atezolizumab-TCO. The authors pre-injected atezolizumab-TCO, and after 24 and 48 h, it was followed by [^99m^Tc]TcHYNIC-PEG11-Tz (specific activity of 9.25 MBq/µg) for an in vivo click reaction. The subjects receiving the imaging agent in this way benefitted from the shortened exposure to radioactivity for such long circulating antibodies. The pharmacokinetic profile of [^99m^Tc]TcHYNIC-PEG_11_-Tz showed a rapid distribution into tissues and renal and hepatobiliary clearance, whereas the uptake in the other organs remained low (<1 %ID/g for the 30 min, 2 h, and 6 h p.i. time points). 

**Figure 4:**
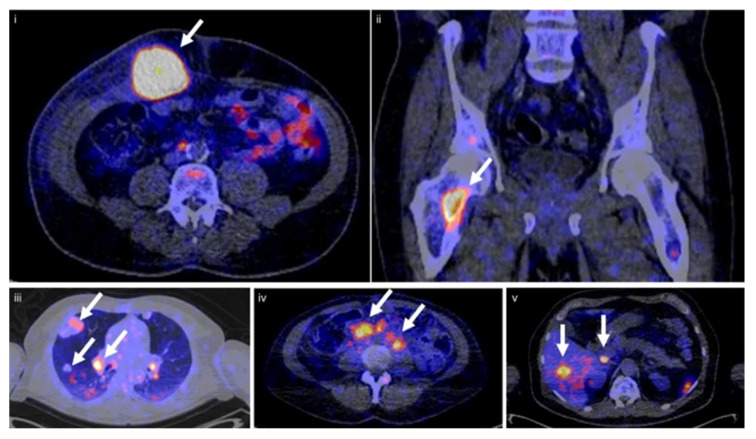
Tumor uptake of [^89^Zr]Zr-sucDf-atezolizumab in cancer patients. Examples of PET/CT images of four patients illustrating the [^89^Zr]Zr-sucDf-atezolizumab tumor uptake in five different locations on day 7 post-injection (white arrows indicate tumor lesions; PET scans were performed once per patient and time point). Images (**i**) and (**ii**) are from the same patient, whereas images (**iii**), (**iv**), and (**v**) are from separate patients. The figure is reproduced with permission from [37].

The first clinical study using [^89^Zr]Zr-sucDf-atezolizumab was performed by Bensch et al. to monitor biodistribution and to evaluate the potential of the radiotracer to predict the response to PD-L1 blockade in patients (Figure 4) [37]. In this study 22 patients—with either bladder cancer, NSCLC, or triple-negative breast-cancer—were administered [^89^Zr]Zr-sucDf-atezolizumab and underwent PET/CT imaging for up to four time points (0, 2, 4, and 7 days). These images revealed low uptake in the brain, subcutaneous tissue, muscle, compact bone, and lung (constant SUV_mean_ in a range of 0.5–1.5 after 2 d), whereas the uptake in the intestines, kidneys, and liver was higher due to antibody metabolism and elimination (constant SUV_mean_ in a range of 4–5 after 2 d). At days 4 and 7 p.i., non-malignant lymph nodes were delineated for most patients, and the spleen was clearly visualized. All of the metastatic lesions were detected (overall geometric mean SUV_max_ of 10.4), a stabilization of the SUV_max_ values of the tumor lesions and tumor-to-background ratios (lung and bone metastases) was noted at day 7 p.i., and, at the same time point, the most favorable tumor-to-blood ratio was achieved. Among multiple patients, heterogeneous intratumoral tracer uptake was observed, which was confirmed by the autoradiography of two tumor samples. The internalization rate of [^89^Zr]Zr-sucDf-atezolizumab in vitro on two tumor cell lines was high in comparison to the internalization rate in healthy peripheral blood mononuclear cells. The complete response (CR), partial response (PR), and stable disease (SD) were noted for three, four, and eleven patients, respectively. Four patients progressed when evaluated at the first CT (6 weeks). The objective response rate was 56%, 11%, and 25% for bladder cancer, NSCLC, and triple-negative breast cancer patients, respectively. A positive correlation was noted between the [^89^Zr]Zr-sucDf-atezolizumab tumor uptake (also related to the target lesion size) and the best tumor response category. The uptake of [^89^Zr]Zr-sucDf-atezolizumab was also related to the change in the size of the lesions. 

Xu et al. developed an antibody-based PET tracer for the imaging of PD-L1 in combination with a therapeutic approach [41]. They utilized the fully human IgG1 antibody MX001 NOTA to allow for the labeling with ^64^Cu. FACS experiments revealed the high binding affinity (5.40 ± 2.30 nM) of ^Nat^Cu-NOTA-MX001 to PD-L1. In a mouse xenograft model with a PD-L1-positive and PD-L1-negative tumor, a consistent tumor uptake was observed, which peaked at 62 h p.i., with a tumor-to-muscle ratio of 62.1 ± 23.3. Accumulation in the liver dominated at early time points and decreased at later time points, indicating clearance through the hepatobiliary system (tumor-to-liver ratio of 3.18 ± 1.06 at 62 h p.i.). The tracer distinguished between the PD-L1-positive and -negative tumor, whereas [^18^F]FDG as a control showed no significant differences in the uptake between both tumors. To pursue an immunotherapeutic approach, mice were administered [^18^F]FDG, and PET/CT scans were acquired ten days after a two-week-long treatment with MX001. The PD-L1 tumors shrank significantly compared to their PD-L1-negative control groups (442.8 ± 18.0 mm^3^ vs. 2523.4 ± 139.9 mm^3^), which indicates a tumor growth suppression of 88%. The PET/CT images of [^18^F]FDG after the treatment supported these findings. The uptake in the PD-L1-positive tumor was higher before the treatment (8.2 ± 2.3 %ID/g) compared to that after the treatment (1.5 ± 0.3 %ID/g), which proves the therapeutic effects of MX001.

Kikuchi et al. studied the upregulation of surface PD-L1 expression in tumors upon radiotherapy monitored via non-invasive immunoPET/CT with a ^89^Zr-labeled anti-PD-L1 monoclonal antibody ([^89^Zr]Zr-DFO-PD-L1 mAb) [42]. The authors treated two different cell/tumor models (MEER and B16F10) subsequently with two different radiotherapy patterns (2 Gy × 4 and 2 Gy × 10). First, in vitro irradiations (single fractions of 2 or 10 Gy) showed significant increases in the PD-L1 levels in both cell lines. Biodistribution and PET/CT imaging (at 48 h p.i.) three days after the completion of radiotherapy (2 Gy × 4) in tumor-bearing mice showed higher tumor-to-blood/muscle ratios in the irradiated (IR) B16F10 tumor compared to those in the non-irradiated (non-IR) tumor, whereas these effects were not observed for the MEER model. In a second experiment, MEER xenograft-bearing mice received radiotherapy (2 Gy × 10) in combination with the administration of an anti-PD-1 antibody or isotope antibody. The tumor uptake and tumor-to-blood/muscle ratios were twofold higher for the IR xenografts in comparison to those for the non-IR xenografts. On day four of the tracer injection, a treatment response to the anti-PD-1 antibody and radiotherapy was observed: the IR/anti-PD-1 mAb-treated tumor volume decreased (tumor volume approx. 80 mm^3^) compared to that of the non-IR/anti-PD-1 mAb and IR/isotype mAb (both approx. 150 m^3^) and non-IR/isotype mAb (approx. 280 m^3^) treatments. Further, the PD-L1 levels on the MEER and B16F10 tumors at days 0, 1, 2, and 3 after the completion of radiotherapy (2 Gy × 4) were determined by flow cytometry. For the MEER tumors, a significant PD-L1 increase was observed at days 0 and 1, which decreased back to an untreated level by day 3. In contrast, the B16F10 tumors showed a significant PD-L1 upregulation until day 3, which remained at this level. These findings are in accordance to the acquired PET/CT images, demonstrating that imaging can discriminate between upregulated and unaffected PD-L1 levels. In summary, external, fractionated radiotherapy induces PD-L1 upregulation, which can be monitored non-invasively using [^89^Zr]Zr-DFO-PD-L1.

Li et al. modified an antibody—consisting of one Fc tail fused with two single domains—with a lower molecular weight (79.6 kDa) than usual antibodies [43]. This antibody, named KN035, exhibits a high affinity to human PD-L1 but does not cross-react with murine PD-L1. PET imaging and ex vivo biodistribution studies with [^89^Zr]Zr-Df-KN035 revealed the highest tumor uptake at 24 h p.i. and a constant tumor-to-muscle ratio of approx. 7.3 up to 120 h after injection. At 120 h p.i., the accumulation in the blood decreased more rapidly compared to the non-target tissues and the tumor, resulting in an improved tumor-to-blood ratio at this time point (1.10 ± 0.12). In another study, Li et al. evaluated the influence of the epidermal growth factor (EGFR) tyrosine kinase inhibitors (TKIs) on tumor growth and PD-L1 expression [44]. A treatment with EGFR TKIs for patients who suffered from NSCLC prolonged their survivability, but the consequences for the tumor environment after such a treatment remain unclear. It is supposed that EGFR-TKIs, such as gefitinib, downregulate the PD-L1 expression in the tumor microenvironment [83]. Therefore, tumor-bearing mice were treated with a high dose of gefitinib for 14 consecutive days or with a low dose for 21 consecutive days. After these treatments, [^89^Zr]Zr-Df-KN035 was administered to these mice to acquire PET images and to perform ex vivo analysis. The tumors grew statistically slower for the high dose treatment, but this was not the case for mice receiving the lower dose treatment. The tracer accumulated significantly less for the high-dose group compared to its control, while the low-dose treatment did not significantly influence the uptake of [^89^Zr]Zr-Df-KN035 after treatment. 

Zhao et al. published an antibody-based radiotracer for the detection of PD-L1 via Cerenkov luminescence imaging (CLI) [45]. This imaging method is inferior in terms of spatial resolution, biological penetration, and signal specificity compared to the PET and SPECT imaging methods but offers a cheaper and simpler operation, allowing for a higher throughput, which can be beneficial for preclinical studies. Therefore, a PD-L1 monoclonal antibody was labeled with ^131^I, which showed a dissociation constant (K_D_) of 1.069 nM. [^131^I]I-PD-L1-mAb was studied in four different mouse models with varying levels of PD-L1 expression. For the highest PD-L1-expressing tumor, the uptake was quite low at 120 h p.i. (approx. 1.5 %IA/g) in comparison to several non-target tissues at the same time point (blood pool: approx. 2.2 %IA/g, spleen: approx. 2.0 %IA/g, lung: approx. 3.8 %IA/g, kidney: approx. 2.4 %IA/g). All four different xenografts could be visualized by Cerenkov imaging, whereas the highest PD-L1-expressing xenograft exhibited the most favorable tumor-to-background ratios (approx. 7, 9, and 13.5 for 24, 48, and 120 h p.i., respectively). In general, higher PD-L1 expression on xenografts showed higher tumor-to-background ratios, demonstrating the efficient discrimination of different PD-L1 expression levels by CLI.

For a theranostic approach, Pang et al. labeled an anti-PD-L1 monoclonal antibody with iodine-131 to combine fluorescence imaging and therapy. The resulting [^131^I]I-PD-L1 mAb was studied in 4T1-Luc xenograft tumor-bearing mice [46]. At 72 h p.i., the highest accumulation of the antibody was observed in the lung, liver, and spleen, followed by the kidneys and the tumor. Therapy studies with a low PD-L1-expressing cell line in mice (approx. 20% PD-L1 expression in the tumor) showed that a combination therapy (^131^I-PD-L1 mAb and, 96 h later, anti-PD-L1 mAb) could suppress the tumor growth drastically over 30 days (approx. 600 mm^3^) compared to the [^131^I]I-PD-L1 mAb-only therapy (approx. 1000 mm^3^) and the anti-PD-L1 mAb-only therapy (approx. 2100 mm^3^). The authors provided two explanations for the observed synergistic effects of the combined therapy: 1. The radiation possibly induces the upregulation of PD-L1 expression in tumor cells, leading to a higher probability of the recognition and infiltration by the T-cells; 2. The emission of beta minus rays leads to a “crossfire effect”, which suppresses the tumor cells or kills them. 

**Figure 5:**
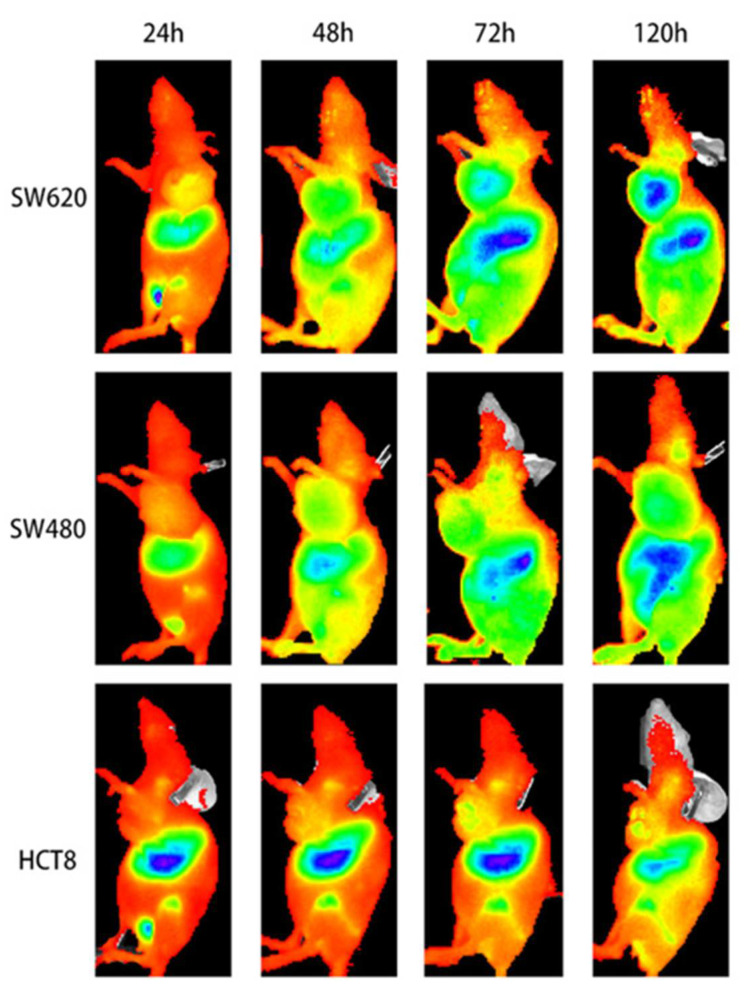
NIR-PD-L1 mAb specifically binds to PD-L1 in human colorectal cancer xenografted mice. Optical images in SW620-, SW480-, and HCT8-grafted mice at different time points. The figure is reproduced with permission from [47].

Zhang et al. conjugated an anti-human PD-L1 monoclonal antibody to the near-infrared dye Licor 800 to generate an optical imaging tracer for PD-L1 (Figure 5) [47]. NIR-PD-L1-mAb was tested in mice bearing colorectal cancer cells. The tracer accumulation positively correlated with the expression levels of PD-L1 in the tumors. As expected, the tumor-to-blood ratios declined with the decreasing PD-L1 expression levels in tumors (5.05 ± 0.36, 3.70 ± 0.10, and 2.99 ± 0.05, respectively).

#### 2.3.2. Heavy Chain Antibodies (HCAb)

Heavy chain antibodies resemble conventional IgG antibodies, but they lack two heavy chains (V_H_) and therefore consist only of two light chains (V_L_). As they still are able to bind to antigens, HCAbs have so far only been found in cartilaginous fishes and in camelids. They bind as specifically as regular antibodies and are in some cases more robust, with their smaller size facilitating the transformation into bacterial cells for bulk production [84,85,86]. 

Huang et al. developed an ^124^I-labeled heavy chain antibody for the PET imaging of PD-L1 [48]. An anti-hPD-L1 HCAbs named Nb6 was labeled with ^125^I, which showed a high binding affinity to PD-L1 (K_D_ = 2.19 nM). Nb6 labeled with ^124^I was used for PET imaging in tumor-bearing mice. The tumor uptake was two-fold higher at 24 h p.i. compared to that at the 48 h p.i. time point, and the opposite was true for the tumor-to-blood and tumor-to-muscle ratios at these time points (T/B ratios of 0.83 ± 0.06 and 1.01 ± 0.12 at 24 and 48 h p.i., respectively; T/M of ratios of 3.61 ± 0.5 and 4.85 ± 0.37 at 24 and 48 h p.i., respectively), indicating the clearance from blood over time.

The same group generated a NOTA conjugate of Nb6 for radiolabeling with ^64^Cu (specific activities in the range of 14–16 GBq/µmol) [49]. [^64^Cu]Cu-NOTA-Nb6 demonstrated a similar binding affinity to PD-L1 (K_d_ = 3.60 nM) as the iodinated version (see above). The uptake of [^64^Cu]Cu-NOTA-Nb6 in the tumor-bearing mice gradually increased in the tumor and non-target tissues over time, achieving the most favorable tumor-to-muscle ratio (approx. 5) between 20 and 38 h p.i.. The limitations of this study are the low spatial resolution of the acquired PET images, the missing blocking studies, and the vague in vivo evaluation. 

#### 2.3.3. Fab Fragments

Fab fragments are engineered antibodies with a separated Fc part cleaved by proteolysis. The omission of the Fc part avoids induced cytotoxic effects, which is highly desirable for immune checkpoint therapy. Since the antigen-binding surface remains unaffected, Fab fragments provide full binding specificity due to their three-fold lower molecular weight compared to conventional antibodies, resulting in better pharmacokinetics for tissue penetration, such as shorter circulation half-lives and fewer undesirable background signals [87,88].

These favorable properties of Fab fragments led to the development of Fab-based radiotracers [87] and inspired Wissler et al. to design such a PET tracer for the imaging of PD-L1 [50]. Therefore, they constructed the Fab fragment by sequence coding from variable regions previously reported for the avelumab antibody and introduced possible sites for chelator conjugation by the amber suppression-mediated genetic incorporation of p-azido phenylalanine. After the conjugation of the NOTA-chelator by a strain-promoted azide-alkyne cycloaddition (SPAAC) reaction, the authors confirmed the binding to PD-L1 in vitro by an ELISA assay (EC_50_ of 0.72 nM for the modified Fab fragment vs. 0.78 nM for the wildtype). This Fab fragment was labeled with ^64^Cu and studied in immune-deficient non-tumor bearing mice. It took 45 min until the signal/noise ratio in PET became stable and the background signals were cleared. During all of the PET scans, a high uptake in the kidneys and urinary bladder was observed, confirming renal clearance and excretion. At 45 min p.i., ^64^Cu-NOTA-αPD-L1 also accumulated in PD-L1-rich tissues such as the brown adipose tissue and spleen. These results confirmed the hypothesis that PD-L1 is not only expressed in extra lymphatic organs [26,89] but also in secondary lymphatic organs [26] and that brown adipose tissue is influenced by the immune system [34,35]. The low accumulation of the control Fab fragment and successful blocking studies demonstrated the specific binding of [^64^Cu]Cu-NOTA-αPD-L1 to PD-L1. Nevertheless, experiments in tumor-bearing mice are missing and are required to investigate the ability of the Fab radiotracer to image PD-L1 overexpression in tumors.

#### 2.3.4. Nanobodies

Nanobodies (Nb) consist of a single monomeric variable antibody domain and are the smallest known engineered antibodies fragments that are still capable of selectively binding to antigens. With molecular weights of 12–15 kDa, the nanobodies weigh only a tenth of the conventional antibodies, resulting in a more rapid tumor uptake, a faster blood clearance, and a higher tumor penetration. Nanobodies can be engineered from heavy-chain antibodies found in camelids (V_H_H fragments), cartilaginous fishes (V_NAR_ fragments), or from IgG antibodies found in mice and humans using genetic engineering techniques [84,87,90].

Lv et al. developed the anti-PD-L1 nanobody Nb109 with a molecular weight of approx. 14 kDa and conjugated it with p-SCN-Bn-NOTA at the nanobodies’ lysine sites, obtaining a mixture of single- and double-NOTA species [51]. Surface plasmon resonance studies revealed a strong binding of Nb109, [^68^Ga]Ga-NOTA-Nb109, and NOTA-Nb109 to recombinant human PD-L1. In tumor-bearing mice [^68^Ga]Ga-NOTA-Nb109 showed the highest tumor-to-muscle ratio (11.03 ± 0.36) at 1 h after injection, whereas the PD-L1-negative control tumors were not visualized at any time point. For the blocking studies, the previously described antibody KN035 was injected one day prior to the tracer injection, revealing similar tumor uptakes of the tracer either with the radiotracer only or pretreated with KN035. The authors suspect different binding epitopes of Nb109 and KN035, so further experiments with another blocking agent would be useful.

Broos et al. pursued another nanobody-based approach, developing a library of thirty-seven nanobodies [52]. Conducting surface plasmon resonance, four nanobodies (C3, C7, E2, E4) were selected which showed high binding to PD-L1 in the low nanomolar range (0.5–17.0 nM). These nanobodies were radiolabeled by complexation with ^99m^Tc-tricarbonyl at their His-tags and were subsequently injected in naïve wild type and PD-L1 knock-out mice to monitor biodistribution. After 1 h, SPECT/CT images were acquired, which revealed high uptake in the kidneys and bladder—proving the kidney retention and urinary excretion of nanobodies—along with low liver uptake in both mouse models. Ex vivo analysis showed an uptake in the lungs, heart, thymus, spleen, lymph nodes, and brown adipose tissue of all the nanobodies. The two best performing tracers, C3 and E2, were chosen for in vivo studies in mice bearing PD-L1 knock-in and PD-L1 knock-down tumors. SPECT/CT images acquired at 1 h p.i. and subsequent ex vivo analysis revealed, contrary to the expectations, that both nanobodies showed the highest accumulation in the tumors derived from the PD-L1 knock-down cells. To obtain further PD-L1-negative tumors, PD-L1 was knocked down in the same cell line using CRISPR/Cas9 technology. In the renewed model tumor, an uptake at the periphery of PD-L1-positive tumors (approx. 1.6 %IA/g for C3) and a lower uptake for PD-L1-negative tumors (approx. 1.1 %IA/g for C3) were observed.

**Figure 6:**
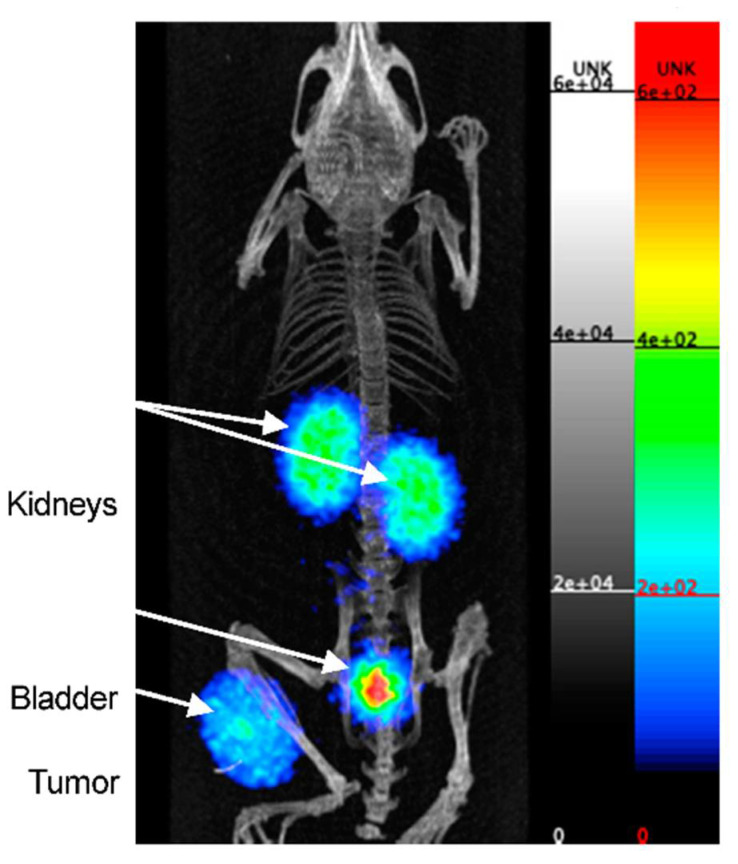
PET/CT image of a mouse bearing a hPD-L1POS tumor at 1 h 20 p.i. of the [^68^Ga]Ga-NOTA-(hPD-L1) nanobody, obtained on the β-CUBE PET/CT system. Scale on the PET image is in kBq/mL. Adapted with permission from [53].

More recently, Bridoux et al. pursued a nanobody-based approach for the non-invasive imaging of PD-L1, too [53]. On one hand, they conjugated the human PD-L1 nanobody (hPD-L1) randomly with NOTA at its lysine sites, and on the other hand, they conjugated it site-specifically using Sortase-A-mediated transpeptidation in order to control conjugation. This approach was used to specify whether a conjugation at the hypervariable lysine regions would alter the binding affinity due to the lysine residues belonging to one of the three complementary determining regions (CDRs). The ^68^Ga-labeled and the randomly and site-specific conjugated nanobodies showed, in a cell saturation binding assay with a transduced hPD-L1-positive cell line, similar dissociation constants for both conjugates (K_D_ = 0.8 and 1.2 nM, respectively). Both tracers were studied in a mouse xenograft model bearing a PD-L1-negative and -positive tumor. The accumulation in the hPD-L1-positive tumors was five times and six times higher than that in the hPD-L1-negative tumors for the randomly and site-specifically conjugated tracers, showing no significant differences between both tracers. No other non-target tissues showed remarkable tracer uptake, except the kidneys and bladder, indicating a renal clearance pathway. This pharmacokinetic profile was confirmed by favorable tumor-to-blood ratios (5.4 ± 1.5 and 6.3 ± 3.0 for the random and site-specific nanobodies) and tumor-to-muscle ratios (28.0 ± 10.6 and 34.5 ± 13.2 for random and site-specific nanobodies). The PET/CT measurements confirmed the results from the ex vivo analysis, with a low background and high tumor uptake at 1 h 20 min p.i. (Figure 6). As a result, the different conjugation strategies exhibited no significantly altered binding affinities in both the cell and mouse experiments. Due to specific tumor targeting and an excellent pharmacokinetic profile, these nanobodies are worth further evaluation by biological investigation for possible clinical translation in the future.

**Figure 7:**
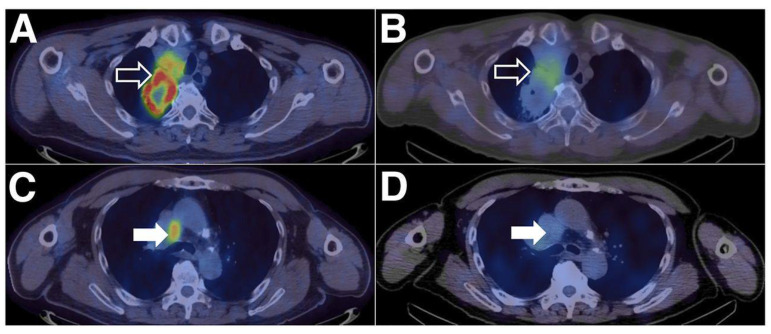
Heterogeneity in PD-L1 expression between primary and nodal sites of disease within the same patient. The right upper lobe tumor (open arrows) shows areas of high [^18^F]FDG uptake (SUVmax = 16.1) on PET/CT (**A**) and [^99m^Tc]Tc-NM-01 SPECT/CT (T:BP = 3.53) (**B**). The mediastinal lymph nodes (closed arrows) show high [^18^F]FDG uptake (SUVmax = 6.3) (**C**) but low [^99m^Tc]Tc-NM-01 activity (T:BP = 1.13) (**D**). The figure is reproduced with permission from [54].

Xing et al. were the first to conduct an early Phase-I study for the non-invasive imaging of PD-L1 utilizing a nanobody [54]. Therefore, the nanobody NM-01 was labeled site-specifically with the ^99m^Tc-tricarbonyl complex. Sixteen patients (11 men, mean age 61.7 years) with histopathologically proven NSCLC were selected, and the PD-L1 expression (0–85%) in primary tumors was determined beforehand by immunohistochemistry. The administered dose of [^99m^Tc]Tc-NM-01 ranged from 255–485 MBq (mean 372 ± 62 MBq), and three patients had to receive a three-fold dose. The safety assessment exhibited that all sixteen patients did not suffer from drug-related adverse events. Planar whole-body and thoracic SPECT/CT scans were acquired at 1 and 2 h p.i., and additional scans were acquired for five patients (10 min, 3 and 24 h p.i.) to calculate radiation dosimetry. The latter demonstrated the highest organ dose in the kidneys (0.036 ± 0.018 mSv/MBq), followed by the bladder (0.026 ± 0.011 mSv/MBq), spleen (0.022 ± 0.011 mSv/MBq), and liver (0.011 ± 0.0025 mSv/MBq). SPECT/CT images demonstrated uptake in physiologically PD-L1-expressing organs, such as the lungs, liver, spleen, and bone marrow, and higher (primary) tumor-to-non-target ratios at the later time point (2 h p.i.): the lung (T:L ratio of 2.69), the blood-pool activity (T:BP ratio of 2.22), and the blood pool for lymph nodes metastases (T:BP ratio of 2.02). The patients with tumors expressing levels of PD-L1 < 1% showed significantly lower tumor-to-blood pool ratios, proving the correlation between T:BP ratios and immunohistochemically determined PD-L1 expression. The patients not only showed an unequal distribution of activity between the primary tumors and their metastases but also within one lesion (Figure 7). One patient also suffered from the accumulation of ^99m^Tc-NM-01 in the bone metastases. In summary, Xing et al., with the first clinical study using a nanobody, provided important insights for the further development of non-invasive imaging agents for PD-L1. The relatively small number of patients, the immunohistochemistry being performed only in primary tumors and not in every patient, and the generally low spatial resolution of the SPECT limited this study.

#### 2.3.5. Affibodies

Affibodies—proteins based on a 58-amino-acid scaffold—are derived from antibodies and mimic their properties such as high binding affinities and specificities. Due to their small molecular weights of approx. 6 kDa, affibodies exhibit high stabilities, the absence of cysteines, and fast blood clearance, which makes them promising candidates for the design of imaging agents [55,91].

These favorable properties motivated Gonzáles Trotter et al. to design the PD-L1 binding affibody [^18^F]AlF-NOTA-Z_PD-L1_1_ [55]. This tracer was improved by the same research group in terms of in vitro and in vivo properties and was published separately three years later [56]. The improved affibody Z_PD-L1_4_ was obtained by the phage display and conjugated site specifically with NOTA. The surface plasmon resonance confirmed a high affinity for both human and rhesus monkey PD-L1 (K_D_ = 0.07 nM). NOTA-Z_PD-L1_4_ was labeled with [^18^F]AlF and ^68^Ga and was studied in tumor-bearing mice with PD-L1-positive and PD-L1-negative tumors. PET imaging revealed renal clearance into the bladder with an extremely high kidney uptake, which was confirmed by ex vivo analysis. Both tracers showed a >25-fold higher uptake in the PD-L1-positive tumors in comparison to that in the PD-L1-negative tumors. Besides the mouse experiments, the biodistribution was monitored in rhesus monkeys, showing similar results for the [^18^F]AlF and ^68^Ga tracer. Fast clearance from the blood, a high kidney uptake, a low liver uptake, and an accumulation in the lymph nodes and spleen (PD-L1-expressing organs [37]) were observed. The estimated dosimetry for human patients showed that both tracers—limited by their kidney accumulation—could be injected up to three times per year for clinical PET scans, according to EU and USA regulations [56].

#### 2.3.6. Peptides

Peptides are, according to the FDA definition, up to 100-amino-acids-long and are connected by amide bonds. They show lower molecular weights compared to the previously described tracer classes. The lower molecular weights enable even faster clearance from the blood pool and non-target organs. Peptides are prone to chemical modifications such as methylation, the introduction of unnatural amino acids, cyclization, and PEGylation, which offers the opportunity to modify them quickly and accurately while improving their in vivo selectivity/stability and pharmacokinetic properties [92].

**Figure 8:**
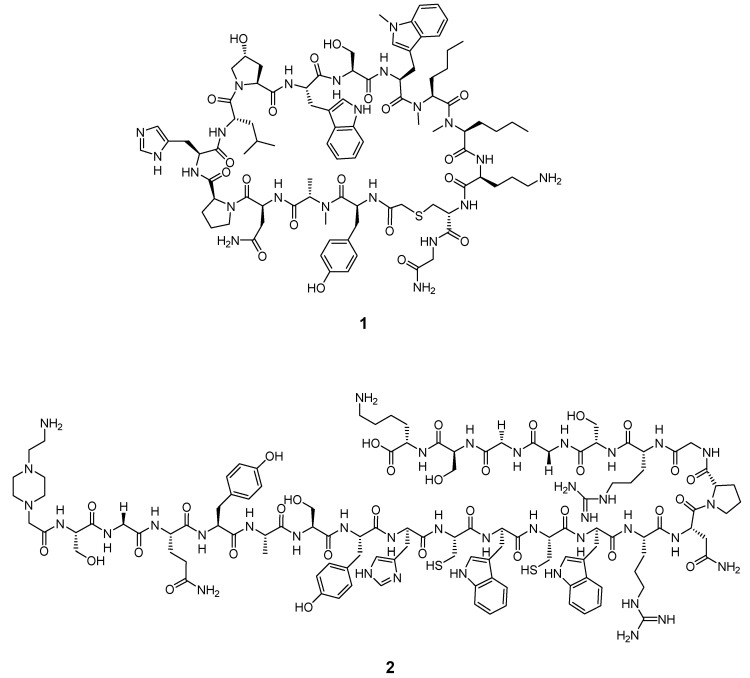
Core structures of the WL12 peptide **1** and native TPP-1 peptide **2**.

Despite the vast amount of reported peptides with high binding affinities to PD-1/PD-L1 [93,94,95], so far, only two lead structures have been chemically modified to allow for radiolabeling and the performance of in vitro and in vivo studies [57,58,59,61]. The lead structures of WL12 and a native TPP-1 peptide are illustrated in Figure 8:

From 2017 to 2020, Nimmagadda and co-workers published three radiotracers (^64^Cu, ^68^Ga, [^18^F]AlF) based on the structure of the 14-mer peptide WL12 [57,58,59]. They conjugated WL12 with DOTAGA at the ornithine residue of the peptide to enable the chelation of the radiometals. In their most recent study, they modified the ornithine residue with 6-[^18^F]fluoronicotinate to generate [^18^F]FPy-WL12 as a radiofluorine-based PET tracer. The radiochemical, in vitro, and in vivo properties of all three tracers are compared in Table 2.

To verify the in vitro binding of these radiotracers, the non-radioactive Cu-version of WL12 (IC_50_ = 2.9 nM) and the nonradioactive ^19^FPy-WL12 (IC_50_ = 26.4–31.7 nM) were evaluated in a FRET assay, revealing that neither the conjugation of DOTAGA and metalation nor the pyridine moiety impaired the binding of WL12 (IC_50_ = 22 nM). DOTAGA-functionalized WL12 was radiolabeled with either ^64^Cu or ^68^Ga. Fluorine radiochemistry involved a nucleophilic substitution of [^18^F]fluoride at a tetrafluorophenyl-nicotinic acid precursor and the subsequent conjugation with WL12. These radiotracers were studied in a mouse xenograft tumor model bearing a PD-L1-positive and PD-L1-negative tumor. Micro-PET/CT imaging demonstrated the fast clearance of all three tracers from the blood pool. The uptake in the tumor and non-target tissues was quantified by ex vivo biodistribution. At 1 h p.i., the highest tumor uptake was observed for [^64^Cu]Cu-DOTAGA-WL12 (14.9 ± 0.8 %ID/g), followed by [^68^Ga]Ga-DOTAGA-WL12 (11.56 ± 3.18 %ID/g) and [^18^F]FPy-WL12 (7.16 ± 1.67 %ID/g). The blocking experiments with nonradioactive WL12 showed significantly reduced tumor uptake for all three tracers. Renal clearance at 1 h p.i. was observed for all three tracers, with a high kidney uptake for [^64^Cu]Cu-DOTAGA-WL12 (34.4 ± 3.1 %ID/g) and [^68^Ga]Ga-DOTAGA-WL12 (64.7 ± 12.1 %ID/g) and a lower kidney uptake for [^18^F]FPy-WL12 (~12 %ID/g). Additionally, a certain liver uptake was noted for [^64^Cu]Cu-DOTAGA-WL12 (24.2 ± 2.5 %ID/g), [^68^Ga]Ga-DOTAGA-WL12 (15.1 ± 7.6 %ID/g), and [^18^F]FPy-WL12 (~32 %ID/g), too. The higher uptake for the ^64^Cu-radioligand can be attributed to hepatobiliary transchelation, which is a commonly observed phenomenon for ^64^Cu-imaging agents [76]. The absence of the hydrophilic DOTAGA chelator in [^18^F]FPy-WL12 increased the liphophilicity of the fluorine radioligand, which likely explains the higher hepatic uptake among all three WL12-based radiotracers. Comparing the tumor-to-muscle ratios at 2 h p.i., an extraordinary high ratio for [^68^Ga]Ga-DOTAGA-WL12 (59.79 ± 16.47) was observed in comparison to that observed for [^64^Cu]Cu-DOTAGA-WL12 (25.6 ± 1.9) and [^18^F]FPy-WL12 (approx. 18). A similar trend was observed for the tumor-to-blood ratios, which were determined at 1 h p.i. 

**Figure 9:**
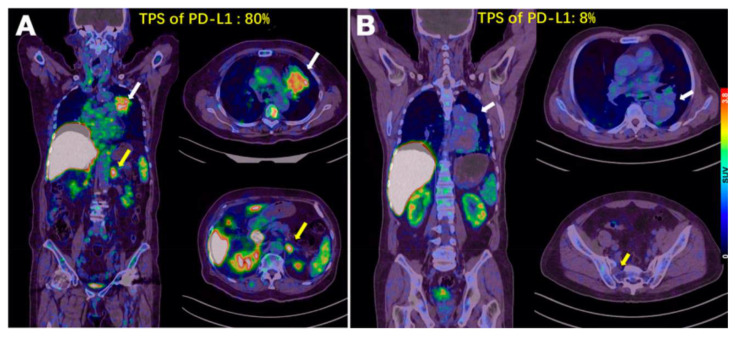
(**A**) An 80-year-old woman with advanced NSCLC and a PD-L1 tumor proportion score (TPS) of 80%. The SUVmax of the primary tumor was 4.87 (white arrow), and that of the left adrenal metastasis was 5.47 (yellow arrow) on [^68^Ga]Ga-NOTA-WL12 PET. (**B**) A 68-year-old man with a PD-L1 TPS of 8%. The SUVmax of the primary tumor in the left lung (white arrow) and right sacral metastasis (yellow arrow) was 1.84 and 0.8, respectively, on [^68^Ga]Ga-NOTA-WL12 PET. Reprinted/adapted with permission from Ref. [96]. 2021 by the Society of Nuclear Medicine and Molecular Imaging, Inc.

As the preclinical data were promising, a first clinical study using [^68^Ga]Ga-NOTA-WL12 was conducted in a small cohort of nine patients with non–small cell lung cancer (NSCLC) [96]. The new radioligand showed uptake mainly in the liver, spleen, small intestine, and kidney. Tumor lesions—particularly in the lung—were clearly visualized, with a tumor-to-lung ratio of 4.45 ± 1.89 at 1 h p.i. The one-hour time point was found to be suitable for image acquisition due to the lack of significant differences in the tumor-to-background ratios between 1 and 2 h. The authors also found a preliminary correlation between tumor uptake (SUVpeak) and the results from the immunohistochemistry of PD-L1 expression (Figure 9). With an administered activity amount of 224 ± 37 MBq, a radiation dose of 4.1 mSv per patient was determined, which is acceptable compared to the dose up to 7.0 mSv for a conventional [^18^F]FDG PET/CT. Therefore, these results are promising for quantifying PD-L1 levels clinically for patient stratification and therapeutic monitoring. 

Inspired by the promising results of the cyclic peptides, Jouini et al. developed a PD-L1-specific macrocyclic peptide NJMP1 based on a structure reported in a patent from Bristol Myers Squibb [63]. The ^68^Ga-labeled peptide was studied on CHO-K1 hPD-L1 cells but showed very low cell binding and internalization rates in comparison to those of the control radiopeptide WL12. These results were confirmed by a non-radioactive cell assay. It was concluded that this patent-derived peptide scaffold is not suitable for radiotracer development and that C-terminal modifications of the macrocyclic peptide interfere with receptor interactions. 

Native peptides exhibit certain advantages over non-natural amino acid-based or macrocyclized peptides: they are scalable at lower costs due to fast access, they have lower immunogenicity, and they have simpler metabolizing patterns. This inspired Kuan et al. to choose the TPP-1 peptide with moderate binding affinity to PD-L1 (K_D_ = 95 nM) for the conjugation with NOTA to allow for [^18^F]AlF and ^64^Cu-labeling [61]. The NOTA conjugate was also PEGylated with a star-like PEG-tetramer to prolong the residence in the body, decrease metabolic degradation, and reduce or eliminate immunogenicity. [^18^F]AlF-TPP-1 and [^18^F]AlF-PEG-TPP-1 both failed to visualize the tumor in tumor-bearing mice, which was attributed to the low molar activities of the tracers (4.05 ± 0.4 and 5.3 ± 0.3 GBq/µmol, respectively). Using ^64^Cu improved the molar activities (36.5 ± 0.5 and 25.8 ± 0.8 for [^64^Cu]Cu-NOTA-TPP-1 and [^64^Cu]Cu-NOTA-PEG-TPP-1, respectively), so both tracers delineated the tumor. The ex vivo analysis of [^64^Cu]Cu-NOTA-TPP-1 revealed a high uptake in the spleen (approx. 34 %ID/g), liver (approx. 25 %ID/g), and kidneys (approx. 70 %ID/g) at 1 h p.i. The tumor uptake of both tracers, [^64^Cu]Cu-NOTA-TPP-1 and [^64^Cu]Cu-NOTA-PEG-TPP-1, peaked at 5 min p.i. (2.0 ± 0.3 and 2.6 ± 0.5 %ID/g, respectively). The non-PEGylated tracer showed a sharper decrease in the tumor-to-muscle ratio, from 10 min to 2 h p.i. (2.2 ± 0.5 to 1.0 ± 0.1), compared to that of the PEGylated analogue (2.2 ± 0.4 at 2 h p.i.), proving the prolonged circulation time by PEGylation. 

**Figure 10:**
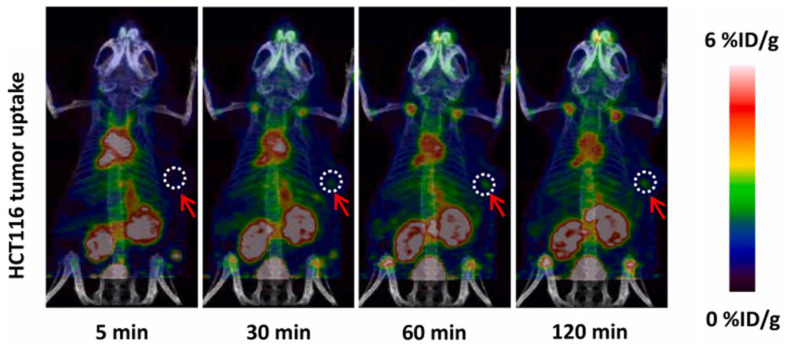
Micro PET/CT images of [^18^F]AlF-NOTA-IPB-PDL1P in a HCT116 mouse tumor model. Reprinted/adapted with permission from Ref. [62]. 2022 Elsevier Inc.

Similarly, Sun et al. also utilized the [^18^F]AlF labeling method for a NOTA conjugate of the new PD-L1-targeting peptide IPB-PDL1P [62]. In vivo microPET studies in a mouse xenograft model showed a high uptake for [^18^F]AlF-NOTA-IPB-PDL1P in the HCT116 and a PC3 tumors, with tumor-muscle ratios of 2.93 and 3.57, respectively, at 120 min p.i. (Figure 10). Biodistribution studies showed a high uptake in the kidneys, gall bladder, and lung and a low tracer uptake in the muscle and brain. 

#### 2.3.7. Small Molecules

**Figure 11:**
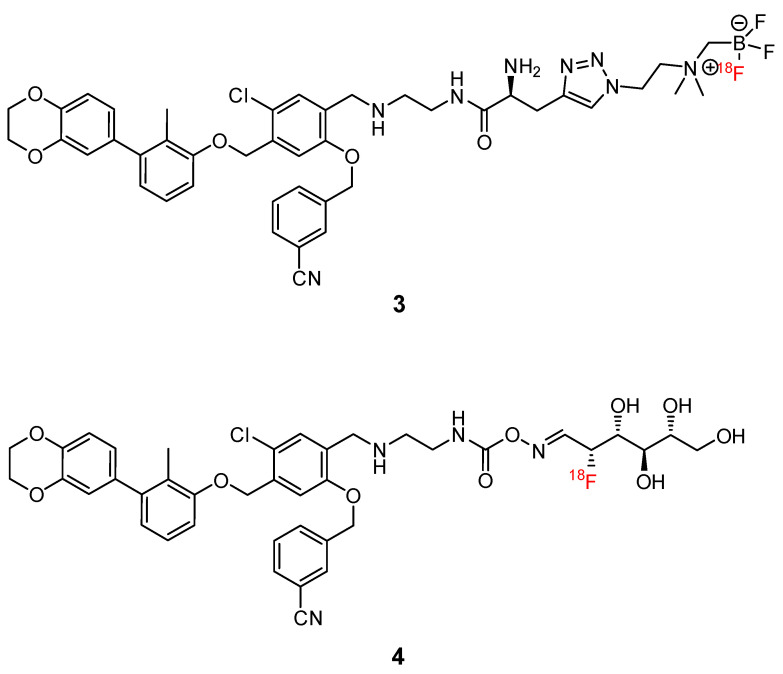
Chemical structures of radiofluorinated small molecule inhibitors [64,65].

Small molecules represent another class of molecules and have already succeeded in tracer development [97]. Small molecules are chemically synthesized organic molecules defined with molecular weights below 500 Da, according to Lipinski [98]. However, many developed small molecule drugs possess molecular weights above 500 Da [99,100]. Due to this low molecular weight, they exhibit a higher oral bioavailability and a faster clearance, and they can be readily upscaled to save costs for institutions, patients, and society as a whole [101]. Despite the report of several organic small molecules inhibiting the PD-1/PD-L1 pathway [102,103,104], only two small molecule-based PET tracers have been published so far (Figure 11) [64,65].

Miao et al. modified the core structure of a molecule disclosed by Bristol-MyersSquibb and labeled it in one step using an ^18^F-^19^F isotopic exchange to generate [^18^F]FLN, (compound **3**) [64]. In a cell saturation binding assay, a dissociation constant (K_D_) of 65.27 nM was obtained, and in a competitive cell binding assay, an IC_50_ value of 50.39 nM was determined. In vivo microPET studies showed a maximum tumor uptake at 15 min after injection. The reduced uptake in a tumor with a lower level of PD-L1 expression and in a tumor pre-blocked with a non-radioactive precursor verified the specific binding of the radioligand. Ex vivo biodistribution analysis at 1 h p.i. revealed higher uptakes in the kidneys and liver compared to the tumor uptake. Due to the lipophilicity of the radioligand, they tried to optimize the radioligand by using the hydrophilic 2-[^18^F]fluoro-2-deoxy-D-glucose ([^18^F]FDG) moiety as a radiolabel and to reduce the overall lipophilicity (compound **4**) [65]. This resulted in a reduced liver uptake and an improved imaging contrast.

#### 2.3.8. Miscellaneous

Inspired by the use of nanotechnology in cancer treatment [105,106,107], Meir et al. conjugated the anti-PD-L1 antibody αPDL1 with gold nanoparticles (GNPs) to develop a theranostic approach for the PD-1/PD-L1 immune checkpoint axis [72]. The gold nanoparticles were supposed to serve as a contrast agent for CT imaging and act as an inhibitor for tumor growth through penetration into the tumor tissue. In addition to the specific αPDL1 antibody, a non-specific IgG antibody was conjugated to PEGylated GNPs to obtain a control species. Flow cytometry studies showed the binding of αPDL1-GNPs to PD-L1 but not for the control species. Both antibody conjugates were injected into tumor-bearing mice, and CT imaging revealed the highest tumor uptake at 48 h p.i., which was approx. 3.3-fold higher than that of the non-specific control agent. αPDL1-GNPs not only accumulated in the tumor tissue but also penetrated intratumorally. Quantified CT images demonstrated a large variation in tumor uptake within the same mouse cohort. With regard to therapy, αPDL1-GNP inhibited tumor growth significantly compared to the control group, which was attributed to the higher T-cell infiltration resulting from a long-lasting immune response treatment. The number of αPDL1-GNPs could be reduced to one fifth of the standard dose of αPDL1 given in clinical care, minimizing the risk of antibody-related adverse side effects while achieving the same results. 

Maute et al. engineered a PD-1 ectodomain by a yeast surface display with a high binding affinity (110 pM) to PD-L1. This engineered protein, HAC-PD1, not only prolonged the survival rates of tumor-bearing mice more successfully than conventional anti PD-L1 monoclonal antibodies, but it also showed favorable properties when used as a ^64^Cu-labeled PET tracer [66]. Based on these results, the same group reported an improved PET tracer two years later [67]. Six radiotracers were designed, varying the chelator (NOTA/DOTA), radiometal (^64^Cu/^68^Ga), and (a)glycosylation. Four of them were investigated by PET imaging and ex vivo biodistribution in tumor-bearing mice. [^64^Cu]Cu-NOTA-HAC-PD-1 showed longer blood circulation and a higher tumor uptake in tumor-bearing mice (1 h p.i.) compared to its DOTA analogue. Both ^64^Cu variants exhibited a high non-specific uptake in the spleen, glands (head/neck region), and liver. The glycosylation of the ^64^Cu-NOTA tracer ([^64^Cu]Cu-NOTA-HACA-PD1) eliminated the glandular signal in the head and neck, reduced the spleen and hepatobiliary uptake, and led to highly specific accumulation in the PD-L1-positive tumors, but it increased the blood circulation time. The exchange of ^64^Cu by ^68^Ga led to a further reduced hepatobiliary uptake, and both [^68^Ga]Ga-NOTA-HACA-PD-1 and [^68^Ga]Ga-DOTA-HACA-PD-1 showed a highly specific tumor uptake and high tumor-to-muscle ratios at 1 h after injection (12.3 and 15.2, respectively). Despite NOTA forming more stable complexes with ^68^Ga, the in vivo performance of the DOTA version surpassed its NOTA analogue. Considering the lower costs of production and the higher clinical relevance of ^68^Ga compared to those of ^64^Cu, [^68^Ga]Ga-NOTA-HACA-PD1 and [^68^Ga]Ga-DOTA-HACA-PD-1 could be interesting candidates for clinical translation after further improvements.

Adnectines, derived from the 10th type III domain of human fibronectin, represent a class of artificially synthesized proteins which bind with high specificities and binding affinities to biological targets. Due to molecular weights of approximately 10 kDa, they target tissues rapidly, which provides a higher imaging contrast within a shorter timeframe compared to antibodies. The high stability and the absence of disulfide bonds make adnectines promising candidates for the development of non-invasive imaging agents [69,108,109]. 

Donnelly et al. isolated one adnectin by screening a 10th fibronectin type II domain library via an mRNA display with expression in an *E. coli* cell line and by purification with several chromatographic methods [69]. This adnectin, when bound to human and cynomolgus PD-L1, exhibited very low dissociation constants (<35 pM), as determined by surface plasmon resonance. This anti-human PD-L1 adnectin was then modified with DBCO-PEG4-Maleimide to introduce a strained alkyne bond. This was followed by copper-free click reaction with a ^18^F-labeled precursor to generate [^18^F]F-BMS-986192. This construct was studied in a mouse xenograft model with moderate, low, or no PD-L1 expression. The acquired PET images showed a plateau of the tumor uptake at 90–120 min p.i. and a significantly higher uptake in the PD-L1-positive tumor at 2 h p.i. Additionally, renal clearance was observed. Ex vivo analysis revealed a moderate uptake in peripheral organs (the liver, lung, and heart) and tumor-to-muscle and tumor-to-blood ratios of approx. 11.5 and 2.5, respectively. In healthy cynomolgus monkeys, the tracer showed accumulation in the PD-L1-positive spleen (spleen-to-muscle ratio of 12:1), which decreased upon co-administration of unlabeled BMS-986192 (spleen-to-muscle ratio of 1.24:1). Based on these data, dosimetry estimations showed that the kidneys are the dose limiting organs. The estimated dose for human subjects was calculated to be 0.22 mSv/MBq, which equals a single administration of 228 MBq of [^18^F]F-BMS-986192 to an average human subject.

Stutvoet et al. showed that [^18^F]F-BMS986192 differentiates the levels of membrane-expressed PD-L1 in four different PD-L1-expressing cell lines using flow cytometry [71]. Additionally, in mice bearing xenografts of these cells, a positive correlation between tumor uptake and PD-L1 expression levels was found. The treatment with proinflammatory cytokine IFNγ induces the in vitro upregulation of membrane PD-L1, which became evident through the higher uptake of [^18^F]F-BMS986192 (24 h after INFγ treatment). In tumor-bearing mice receiving three doses of IFNγ, however, only a minor increase in the tumor uptake of the tracer was observed. In the next experiment, the authors treated PD-L1-positive cells with selumetinib, a mitogen-activating protein kinase inhibitor, resulting in a 50% decrease in PD-L1 expression. Tumor-bearing mice that were treated with selumetinib (1 day with 10 mg/kg) were administered [^18^F]F-BMS986192. However, the PET imaging and ex vivo analysis showed that they did not respond to that treatment. 

**Figure 12:**
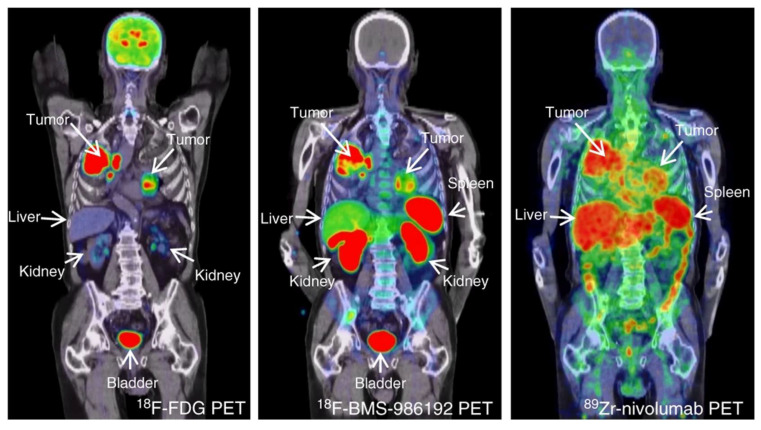
PET imaging of a patient with non-small-cell lung cancer [18]. (**Left**): Image showing the [^18^F]FDG PET scan, which shows the high glucose metabolism of tumors in both lungs and mediastinal lymph nodes. (**Middle**): [^18^F]F-BMS-986192 PET (145.7 MBq, 1 h post-injection (p.i.)) and (**Right**): ^89^Zr-labeled nivolumab PET (37.09 MBq, 162 h p.i.) demonstrate the heterogeneous tracer uptake within and between tumors. The figure is reproduced with permission from [18].

Because of the promising preclinical results, Niemeijer et al. conducted the first clinical in-human study of [^18^F]F-BMS986192 in combination with the PD-1-addressing antibody tracer [^89^Zr]Zr-nivolumab (Figure 12) [18]. The study involved thirteen patients who suffered from NSCLC and included three whole-body PET scans: 1. 90 min after the injection of [^18^F]F-BMS986192-addressing PD-L1 in tumor lesions (overall day 0); 2. 162 h after the injection of [^89^Zr]Zr-nivolumab-addressing PD-1 in tumor-inflating lymphocytes located in the tumor microenvironment (overall day 7); 3. 160 h after the injection of [^89^Zr]Zr-nivolumab and nivolumab to assess the potential response rate to an immunotherapy (overall day 19). For both tracers, no tracer-related adverse effects of grade 3 or higher were observed. [^18^F]F-BMS986192 and [^89^Zr]Zr-Df-nivolumab both had high accumulation in the spleen (PD-1- and PD-L1-positive lymphocytes and dendritic cells), and both showed hepatobiliary uptake (catabolism); additionally, [^18^F]F-BMS986192 was excreted biliary/renally, and [^89^Zr]Zr-Df-nivolumab was excreted gastrointestinally. Both agents accumulated slightly to moderately in the lung and bone marrow but did not do so at all in healthy brains. [^18^F]F-BMS986192 and [^89^Zr]Zr-Df-nivolumab visualized in mean 3.5 lesions and 3.2 lesions per patient, respectively, whereas the lesional uptake of both radiotracers revealed a positive correlation (R_s_ = 0.68) to each other, but this was mainly driven by lesions with a low uptake of [^18^F]F-BMS986192 accumulated heterogeneously in different lesions and also heterogeneously within one lesion, underlining the clinical need of a non-invasive imaging agent. Two patients suffered from brain metastases, which could be partially delineated by both tracers but were generally visualized with lower SUV values compared to the non-brain lesions. This contributed to the central nervous system (CNS)/ blood-brain-barrier and/or the varying PD-1/PD-L1 expression. The immunohistochemical results of non-CNS lesions (determination of PD-1/PD-L1 expression) correlated well with the median SUV_peak_ for [^18^F]F-BMS986192 and [^89^Zr]Zr-Df-nivolumab: a higher SUV_peak_ for lesions with ≥50% tumor PD-L1 expression compared to lesions with <50% tumor PD-L1 expression for [^18^F]F-BMS986192 (8.2 vs. 2.9), and a higher SUV_peak_ for tumors with aggregates of PD-1 for [^89^Zr]Zr-Df-nivolumab (7.0 vs. 2.7). Five of thirteen subjects responded to treatment with nivolumab, whereas the response rate correlated with PD-1 expression but not with PD-L1 expression. However, for both tracers, a positive correlation between the tumor uptake (only for lesions >20 mm) and response rate was demonstrated. The SUV_peak_ values were higher for the responding compared to the non-responding patients for [^18^F]F-BMS986192 (median SUV_peak_, 6.5 vs. 3.2) and [^89^Zr]Zr-Df-nivolumab (median SUV_peak_, 6.4 vs. 3.9). This combined clinical study with a rapidly and slowly accumulating radiotracer (addressing PD-L1 and PD-1, respectively) revealed the heterogeneity of PD-L1 expression between and within tumor lesions, a positive correlation between tracer uptakes and PD-1/PD-L1 expression levels, and a relationship between the SUV_peak_ and the response rate for both tracers.

To further evaluate the behavior of [^18^F]F-BMS986192 in patients, Huisman et al. conducted a clinical study with thirteen patients to quantify the kinetics of the tracer by identifying an optimal pharmacokinetic model [70]. 

Natarajan et al. engineered a protein binder for PD-L1 using a 12 kDa human fibronectin typ-3 domain (FN3) scaffold, which was conjugated with DOTA [68]. The resulting conjugate was studied in tumor-bearing mice (hPD-L1 non-blocked tumor, hPD-L1 pre-blocked tumor, and PD-L1-negative tumor) using PET/CT imaging. Since [^64^Cu]Cu-DOTA-FN3hPD-L1 is mainly secreted by the kidneys, a high renal uptake was observed for all three models 24 h after injection. Significant differences in the tumor uptake between the blocked and non-blocked mice were already noted at 1 h p.i. At 24 h p.i., the tumor uptake reached a plateau, whereas the tumor uptake in the blocked and PD-L1-negative mouse model remained low throughout the whole-time span. In another tumor model using naturally expressing MDA-MB-231 cells, the tumor was clearly delineated starting from 1 h p.i. and reached the highest uptake at 24 h p.i., which was significantly higher compared to that of the corresponding tumors blocked with the nonradioactive compound. Ex vivo biodistribution corroborated these results. 

## 3. Discussion and Outlook

Over the past 20 years, immune checkpoint inhibitor therapy has developed into an important tool for treating cancer. However, the response rates are relatively low and require careful decisions from the treating physician as to whether or not to implement immune checkpoint inhibitor therapy. To date, the decision is based on biopsies, which are not only a burden to patients but also lack the ability to address the heterogeneity of the expression levels of PD-1 and PD-L1 in the tumor microenvironment. Therefore, radiotracers for PET, SPECT, and optical imaging have been developed to provide a whole-body scan, which allows for a more precise therapy decision. While radionuclides are used as a signaling flag in PET and SPECT radiotracers, in OI tracers, fluorophores are utilized. Both techniques are able to provide a whole-body scan, but radiotracers expose the patient to ionizing radiation in contrast to OI tracers. However, the acquisition time for PET and SPECT is in the range of seconds to minutes, while it is up to one hour for optical imaging [110]. Additionally, there is no depth limit for PET and SPECT, and the spatial resolution for clinical PET is ca. 5 mm and below and that of clinical SPECT is ca. 1 cm and below. For optical/ fluorescence imaging, the spatial resolution depends on the imaging depth. In the case of reflectance imaging, the limit is less than 1 cm, and for fluorescence tomographic techniques up to 10 cm, the spatial resolution can be estimated approx. as 1/10 of the imaging depth. PET has a 10- to 100-fold higher sensitivity than SPECT and a 100- to 1000-fold higher sensitivity than OI [111]. Therefore, the detection of PD-L1-positive lesions may not always be possible with OI agents, especially when the PD-L1 expression is lower. On the other hand, optical imaging enables fluorescence-guided surgery, which not only ensures the complete resection of the tumor but also reduces the risk of removing healthy tissue and increases accuracy. Additionally, this method can reduce the operative time and the need for second-look surgeries [112]. Du et al. were able to prove this in the context of the PD-1/PD-L1 immune checkpoint using a fluorescence-labeled PD-1 antibody [25]. However, optical imaging agents can suffer from dye-induced photoinstabilities, and the attachment of a dye to the pharmacologically active structure (i.e., pharmacophore) can negatively influence the biological activity. Especially for small molecule- or peptide-based conjugates, the attachment of high molecular weight dyes can significantly increase the molecular weight of the considered tracer. In contrast, the attachment of a small radioactive label such as ^11^C or ^18^F to the biologically active structure reduces the likelihood of impacting the biological activity dramatically. The combination of PET or SPECT with OI into dual-modal imaging tools can often reduce the disadvantages of either imaging technique [113]. 

**Figure 13:**
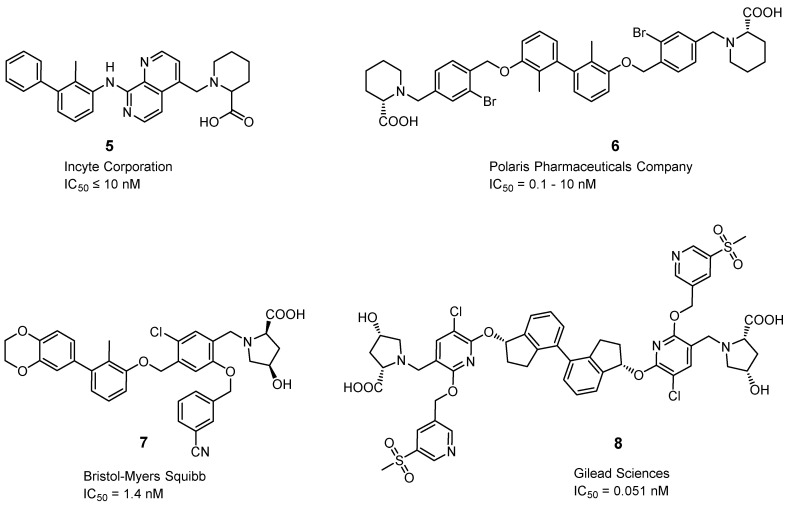
Examples of small molecule inhibitors targeting PD-L1, as disclosed by pharmaceutical companies [104,118,119,120].

A large number of antibodies and derived minibody-, affibody-, and nanobody-based radiotracers have been developed, all of which exhibit advantageously high accumulation in tissues. However, these molecules possess a relatively high immunogenicity, causing potentially adverse immunological effects such as cytokine storms. On the other hand, peptides and non-peptide small molecules accumulate faster in tumor tissue and allow for the imaging of the patient on the same day. The clearance of the tracer is also rapid and within the favorable time range of minutes to hours. Additionally, these tracers are more readily available due to the ease of GMP production. In recent years, a vast number of peptide-based and small molecule inhibitors for the PD-1/PD-L1 blockade have been disclosed [114,115,116]. However, the development of small molecule inhibitors has focused only on the targeting of PD-L1 due to the low sequence homology between murine PD-1 and human PD-1, thus preventing the development of PD-1 inhibitors. Small molecules have the advantages of high tissue penetration and the crossing of cellular membranes, which allow for the reaching of target tumor sites that are inaccessible for larger molecules [117]. In addition, they are generally more stable and possess low immunogenicity, preventing adverse effects. Figure 13 shows selected examples of small molecule structures with high binding affinities toward PD-L1 which are disclosed by pharmaceutical companies [104,118,119,120].

To date, radiopharmaceutical chemistry has rarely employed small molecules for the development of radiotracers for the PET or SPECT imaging of PD-L1 expression in cancer patients [64]. However, small molecule-based radiotracers could overcome certain disadvantages of large biomolecule-based radiotracers such as long circulation times and potential immunogenic adverse effects. They possess favorable pharmacokinetic properties, a fast tissue uptake, and short clearance times, which—together with a high signal-to-noise ratio—allow for imaging within minutes to hours post-injection, resulting in a lower radiation burden for the patient. The biological half-life of small molecules matches the physical half-life of short-lived PET nuclides such as ^15^O, ^13^N, ^11^C, and ^18^F, which, especially in the case of ^18^F, favorably increases the resolution limits of the PET images [121]. So far, only one small molecule PD-L1 inhibitor has been radiofluorinated by employing an ^18^F-^19^F isotope exchange on an alkylammoniomethyltrifluoroborate (AMBF3) moiety (see compound **3** in Figure 11 above) [64]. Because the radioligand was very lipophilic and exhibited a high liver uptake, another more hydrophilic radioligand bearing a 2-[^18^F]fluoro-2-deoxy-D-glucose ([^18^F]FDG) moiety was synthesized from the same parent molecule [65]. 

**Figure 14:**
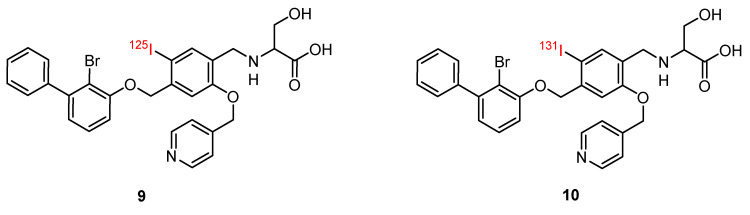
Examples of radioiodinated small molecule PD-L1 inhibitors for diagnostic (**9**) and therapeutic (**10**) applications [125].

The introduction of radioiodine such as iodine-123 for SPECT imaging or iodine-124 for PET imaging offers another option for the radiolabeling of small molecules. Radioiodine isotopes are produced in an iodide form, which allows for radiolabeling via nucleophilic substitution, electrophilic substitution, or isotopic exchange reactions, among other methods [122]. Because iodine is a large halogen, approximately the size of a phenyl ring, the radiolabeling of small molecule PD-L1 inhibitors with radioiodine potentially alters the physicochemical and biological properties, which could have a negative impact on the binding affinities and tumor uptake [123]. Another crucial point which has to be considered is the deiodination of the radioligand that occurs depending on the iodination pattern in the chemical structure [124]. Deiodination can lead to the accumulation of radioiodine in off-target tissues such as thyroid and stomach, thus reducing the image quality [123]. A patent by Feng et al. claimed a series of radioiodinated small molecule PD-L1 inhibitors for the imaging and therapy of PD-L1-positive tumors [125] (Figure 14). 

**Figure 15:**
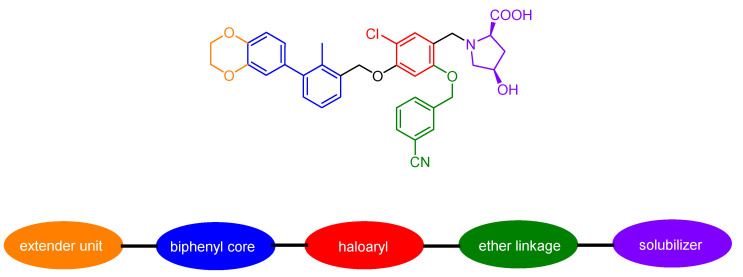
Structural analysis of biphenyl-based small molecule PD-L1 inhibitors [114].

The radiometalation of bioactive molecules in general is performed through the chelation of the metal ion. However, there are a number of challenges in attaching a chelator to a small molecule PD-L1 inhibitor. The first point is attaching a chelator without affecting the binding affinity. Since the biphenyl moiety, the central haloaryl, and the ether building block are essential for the binding affinity, the attachment of a chelator would be possible only at either the extender or the solubilizer unit (Figure 15). Additionally, the chelator should be placed at some distance from the binding site to prevent a negative impact of the binding affinity. The second point to be considered is the alteration of the pharmacokinetic properties, such as biodistribution, lipophilicity, metabolism, and excretion, according to the ADME criteria. For the clinically used FAPα targeting radioligands, the introduction of a water-soluble piperazine linker between the binding moiety and the chelator has been the key for their favorable pharmacokinetics [126,127]. We have adopted this concept for PD-L1-targeting small molecules and introduced the chelator via a hydrophilic linker unit [128]. 

**Figure 16:**
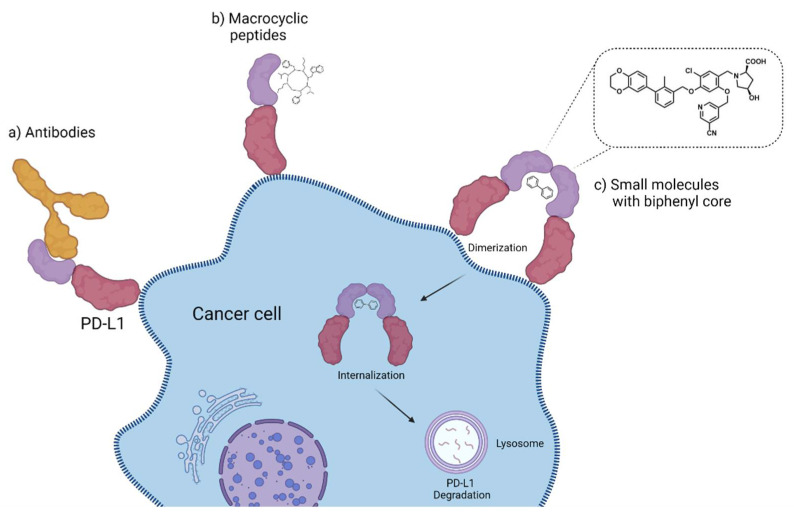
Binding modes of PD-L1-targeting antibodies, peptides, and biphenyl-based small molecule inhibitors [129]. The latter ones show internalization and lysosome-dependent degradation of PD-L1 [132].

In the past years, biological studies have revealed that the binding mode of small molecule inhibitors differs significantly from that of antibodies and peptides (Figure 16) [129]. While both antibodies and peptides bind in a 1:1 molar ratio to PD-L1 and thus prevent the binding of the natural ligand PD-1 in an antagonistic manner, biphenyl-derived low-molecular weight compounds recruit two PD-L1 proteins, leading to the dimerization of the human PD-L1 in vitro. This dimerization was reported to result in the downregulation of the cell surface expression of PD-L1 in tumor cells [130]. Additionally, it was found that the binding of a biphenyl inhibitor to PD-L1 at the early stages of protein maturation suppresses protein glycosylation and prevents the transport of the under-glycosylated form of PD-L1 from the endoplasmic reticulum to the Golgi apparatus [131]. A recent report showed that the internalized PD-L1 is subjected to lysosome-dependent degradation triggered by biphenyl inhibitors (Figure 16) [132]. Based on the unique internalization and degradation mechanism of biphenyl-based small molecule inhibitors, derived radioligands could theoretically offer the possibility for an endoradiotherapy approach with beta- or alpha-emitting nuclides. However, the receptor densities in tumor cells may be too low to carry sufficient amounts of the radiotracer into the tumor in order to achieve an effect. On the other hand, the high upregulation of PD-L1 in other cell types surrounding the tumor offers the possibility to still destroy the tumor cells via long-range beta-emitters, causing a crossfire effect.

In general, PD-1 and PD-L1-targeting radio and optical tracers can offer clinicians a valuable tool for making decisions on immune checkpoint inhibitor therapy. However, the expression of PD-L1 is often low, and off-target effects due to the natural PD-L1 expression in healthy tissues are possible, thus resulting in low tumor-to-background ratios. In addition, the expression levels of PD-L1 do not always correlate with the outcome of the immune checkpoint inhibitor therapy [133], which limits the application of such radio and optical tracers. From the viewpoint of tumor biology, there is still an ongoing search for the ideal biomarker to guide patient stratification and therapy monitoring [134]. 

In summary, radiolabeled antibodies and peptides targeting PD-1 or PD-L1 for PET, SPECT, and optical imaging have undergone extensive preclinical and first-clinical studies, showing their potential as prognostic tools for patient stratification and therapy monitoring. The potential of small molecules for the development of PD-L1-targeting radioligands has not been fully explored, despite their promising favorable physiological properties. A unique PD-L1 dimerization, internalization, and degradation mechanism triggered by biphenyl-based small molecules may offer the possibility for a radionuclide-based endoradiotherapy to treat cancer. Therefore, development of small molecule-based PD-L1 radioligands should receive more attention among radiopharmaceutical scientists and clinicians to benefit cancer patients in the future. 

## Figures and Tables

**Table 2:** Comparison of the radiochemical, in vitro, and in vivo properties of all three WL12 radioligands.

	[^64^Cu]Cu-WL12	[^68^Ga]Ga-WL12	[^18^F]FPy-WL12
Molar Activity	28.5 ± 1.65 GBq/µmol	128 GBq/μmol.	105 ± 54 Ci/mmol
IC_50_ [nM]	2.9	n.a.	37.1
%ID/g at 60 min p.i.	14.9 ± 0.8 (tumor) 34.4 ± 3.1 (kidney) 24.2 ± 2.5 (liver)	11.56 ± 3.18 (tumor) 64.7 ± 12.1 (kidney) 15.1 ± 7.6 (liver)	7.16 ± 1.67 (tumor) ~12 (kidney) ~32 (liver)
Tumor-to-Muscle Ratio, 2 h p.i. Tumor-to-Blood Ratio, 1 h p.i.	25.6 ± 1.9	59.79 ± 16.47	~18
	4.7 ± 1.2	7.56 ± 16.47	~4

## Data Availability

No new data were created or analyzed in this study. Data sharing is not applicable to this article.
